# Spatially resolved transcriptomics reveals genes associated with the vulnerability of middle temporal gyrus in Alzheimer’s disease

**DOI:** 10.1186/s40478-022-01494-6

**Published:** 2022-12-21

**Authors:** Shuo Chen, Yuzhou Chang, Liangping Li, Diana Acosta, Yang Li, Qi Guo, Cankun Wang, Emir Turkes, Cody Morrison, Dominic Julian, Mark E. Hester, Douglas W. Scharre, Chintda Santiskulvong, Sarah XueYing Song, Jasmine T. Plummer, Geidy E. Serrano, Thomas G. Beach, Karen E. Duff, Qin Ma, Hongjun Fu

**Affiliations:** 1 Department of Neuroscience, College of Medicine, Ohio State University, Columbus, OH 43210 USA; 2 Biomedical Sciences Graduate Program, Ohio State University, Columbus, OH 43210 USA; 3 Department of Biomedical Informatics, College of Medicine, Ohio State University, Columbus, OH 43210 USA; 4 UK Dementia Research Institute, UCL Queen Square Institute of Neurology, London, UK; 5 The Steve and Cindy Rasmussen Institute for Genomic Medicine, Abigail Wexner Research Institute at Nationwide Children’s Hospital, Columbus, OH 43205 USA; 6 Department of Neurology, Center for Cognitive and Memory Disorders, Center for Neuromodulation, Ohio State University, Columbus, OH 43210 USA; 7 Department of Biomedical Sciences, Cedars-Sinai Medical Center, Los Angeles, CA 90048 USA; 8 Banner Sun Health Research Institute, Sun City, AZ 85351 USA

**Keywords:** Spatially resolved transcriptomics, Alzheimer’s disease, Vulnerability, Human middle temporal gyrus, Microglia, Oligodendrocytes, Astrocytes, Neurons, Weighted gene co-expression network analyses (WGCNA), Single-molecule fluorescent in situ hybridization

## Abstract

Human middle temporal gyrus (MTG) is a vulnerable brain region in early Alzheimer’s disease (AD), but little is known about the molecular mechanisms underlying this regional vulnerability. Here we utilize the 10 × Visium platform to define the spatial transcriptomic profile in both AD and control (CT) MTG. We identify unique marker genes for cortical layers and the white matter, and layer-specific differentially expressed genes (DEGs) in human AD compared to CT. Deconvolution of the Visium spots showcases the significant difference in particular cell types among cortical layers and the white matter. Gene co-expression analyses reveal eight gene modules, four of which have significantly altered co-expression patterns in the presence of AD pathology. The co-expression patterns of hub genes and enriched pathways in the presence of AD pathology indicate an important role of cell–cell-communications among microglia, oligodendrocytes, astrocytes, and neurons, which may contribute to the cellular and regional vulnerability in early AD. Using single-molecule fluorescent in situ hybridization, we validated the cell-type-specific expression of three novel DEGs (e.g., *KIF5A*, *PAQR6*, and *SLC1A3*) and eleven previously reported DEGs associated with AD pathology (i.e., amyloid beta plaques and intraneuronal neurofibrillary tangles or neuropil threads) at the single cell level. Our results may contribute to the understanding of the complex architecture and neuronal and glial response to AD pathology of this vulnerable brain region.

**Supplementary Information:**

The online version contains supplementary material available at 10.1186/s40478-022-01494-6.

## Introduction

Alzheimer’s disease (AD) is the most common form of dementia in the elderly, affecting 40–50 million people worldwide [1]. AD is pathologically characterized by extracellular amyloid beta (Aβ) plaques, neurofibrillary tangles (neuronal tau aggregates), gliosis induced by activated microglia and reactive astrocytes, and white matter (WM) degeneration possibly induced by dysfunctions in oligodendrocytes [2–6]. The distribution pattern of AD pathology from regions like the medial temporal lobe to the cortex has been extensively studied [7–10]. However, the molecular mechanisms underlying the cell- and region-specific distribution of AD pathology at early stages of the disease are still under-investigated. The transcriptome of the AD brain can pinpoint key differences in disease that may be crucial for elucidating the pathogenesis of AD and for developing disease-modifying therapeutics for the prevention and treatment of AD [11].

Compared with single-cell or single-nucleus (sn) RNA-Sequencing (RNA-Seq) techniques, the recent advent of spatially resolved transcriptomics (SRT) (e.g., 10 × Genomics Visium) allows us to compare transcriptomic profiles between AD and CT subjects without the loss of spatial information [12–17]. This novel technique enables a better understanding of molecular mechanisms underlying the neuropathology of AD within the spatial context. Recent applications of SRT on AD-like mouse models have revealed a plaque-induced gene (PIG) network, an oligodendrocyte gene (OLIG) response, and regionally differentially expressed genes (DEGs) in AD-like mice compared to controls [16, 17]. However, comprehensive SRT profiling of human AD brain tissue and characterization of DEGs associated with variations of AD pathology in vulnerable cortical layers, have not been reported as far as we know.

To fill this knowledge gap, we used the 10 × Genomics Visium platform combined with co-immunofluorescence staining of AD-associated pathological markers to define the spatial topography of gene expression in the human middle temporal gyrus (MTG), a vulnerable brain region in early AD [18]. In this study, we investigated the MTG from human postmortem CT (Braak stages I-II, minimal tau pathology) and AD cases (Braak stages III-IV, moderate tau pathology) [8]. To highlight gene changes associated with early stages of the disease and exclude most downstream gene expression changes that occur in late AD, we did not include AD cases at Braak stages V-VI in this study. We annotated the cortical layers and the WM of AD and CT MTG, identified specific marker genes for five cortical layers and the WM, and identified layer-specific DEGs in human AD compared to CT. Weighted gene co-expression network analyses (WGCNA) [19] of 10,000 highly variable genes in both AD and CT cases revealed eight co-expression gene modules. The co-expression pattern of four gene modules dramatically changed in the presence of Aβ and/or tau pathology, implicating an important role of varying cell types and their co-expression in health and disease. Furthermore, we quantitated the expression of novel DEGs associated with AD pathology in microglia, astrocytes, neurons, and oligodendrocytes using RNAscope HiPlex single-molecule fluorescent in situ hybridization (smFISH) assay. We further validated our study and techniques by quantitating the expression of previously published DEGs [12–15, 20–25]. As far as we know, our study provides first-of-its-kind SRT profiling of human MTG and a unique spatial view of transcriptional alterations associated with two major AD hallmarks, Aβ plaques and pathological tau. Furthermore, this analysis reveals gene perturbations specific to each layer and AD pathology, as well as shared gene-expression perturbations, thus providing additional molecular insights into the regional vulnerability and pathogenesis of AD.

## Methods

### Human postmortem brain tissues

Human fresh frozen brain blocks were provided by the Arizona Study of Aging and Neurodegenerative Disorders/Brain and Body Donation Program at Banner Sun Health Research Institute [26], the New York Brain Bank at Columbia University Irving Medical Center [27], and the Brain Bank & Biorepository at Ohio State University Wexner Medical Center. The demographics of human cases used in this study are listed in Additional file 2: Table S1. These specimens were obtained by consent at autopsy and have been de-identified and are IRB exempt to protect the identity of each patient. Frozen sections (10 μm) were cut from frozen blocks under RNase-free conditions.

### Reagents

Human/murine phospho-tau pSer202/Thr205 (AT8, Cat# MN1020), Tau46 (Cat# 13-6400), Alexa Fluor dye-labeled cross-absorbed donkey secondary antibodies, and TRIzol RNA isolation reagent (Cat# 15596026) were purchased from ThermoFisher Scientific. Goat anti-Olig2 (Cat# AF2418) and GAD1 (Cat# AF2086) polyclonal antibody were purchased from R&D Systems. Rabbit anti-GFAP (Cat# G9269), WFS1 (Cat# 1158-1-AP), and Aβ (Cat# Ab2539) polyclonal antibodies were purchased from Sigma-Aldrich, Proteintech, Abcam, and DAKO, respectively. Rat anti-P2RY12 (Cat# 848002) was purchased from BioLegend. RNAscope HiPlex Ancillary Kit (Cat# 324120) and human-specific RNA probes were purchased from Advanced Cell Diagnostics. TrueBlack lipofuscin autofluorescence quencher (Cat# 23007) was purchased from Biotium. Fluoromount-G Mounting Medium (Cat# 0100-01) was purchased from SouthernBiotech.

### Sample selection and preparation for Visium

Thirty milligrams of brain tissue were homogenized in 500 µl TRIzol RNA isolation reagent. RNA was extracted from each homogenate by following the TRIzol RNA extraction procedure. The RNA quality of each sample was assessed by RNA integrity number (RIN) via Agilent 2200 TapeStation system. The 10 µm sections from the human MTG were mounted within 6.5 mm × 6.5 mm capture areas, which were subjected to the Visium gene expression assay. All samples passed the quality control for cDNA, post library construction, and sequencing set by 10 × Visium Spatial Gene Expression Reagent Kits User Guide (Additional file 3: Table S2).

The time course of the tissue permeabilization for each sample was determined by 10 × Genomics Visium Spatial Tissue Optimization (STO) Kit (Slide Kit, Part# 1000191. Reagent Kit, Part# 1000192). Briefly, sections from selected square areas of the brain tissue were mounted on the capture areas of STO slides. Sections were fixed in the −20 °C prechilled methanol for 30 min and subjected to H&E staining. The 20 × tile image for each sample was obtained by Zeiss Axio Observer microscope. After imaging, sections were permeabilized with permeabilization enzyme for varying amounts of time, and the reverse transcription (RT) was performed directly on the slide using Cy3-nucleotides. After RT, sections were imaged and aligned with H&E images. The best time course was chosen for Vsisum experiment if its image shows the strongest Cy3 signal and the minimum signal diffusion.

### Visium SRT processing

Each optimized human postmortem brain tissue was cryosectioned at 10 µm continuously for five sections and was labeled sequentially as No. 1 to No. 5. The middle section (No. 3, Gene Expression (GE) section) was mounted on the Visium GE slide for Visium profiling, sections No. 1, 2, 4, 5 were used for IF staining of cell-type markers and AD pathology. SRT was performed using the 10 × Genomics Visium Spatial Gene Expression Kit (Slide Kit, Part# 1000188. Reagent Kit, Par# 1000189). The procedures prior to RT are the same as described for STO. For RT, cDNA was synthesized using nucleotides without the label of Cy3. By using Template Switch Oligo, the second strand cDNA (ss cDNA) was synthesized according to cDNA templates captured on the poly T probes. The ss cDNA was then denatured by 0.08 M KOH, washed off, and then amplified using PCR. The cDNA quality control was performed using an Agilent Bioanalyzer high sensitivity chip. After the cDNA concentration was determined, the sequencing library of each sample was constructed using the 10 × Library Construction kit (Part# 1000196). Briefly, optimized cDNA was obtained by enzymatic fragmentation and size selection. P5, P7, i7, and i5 sample indexes and TruSeq Read 2 were added via End Repair, A-tailing, Adaptor Ligation, and PCR. The cDNA library with correct sizes was then selected using the SPRIselect reagent (Beckman Coulter, Part# B23318). To meet the required sequencing depth, cDNA libraries from four samples were pulled in a NovaSeq6000 SP v1.0 flowcell and paired-end sequencing was performed on an Illumina NovaSeq6000 sequencer at the Genomics Services Laboratory at Nationwide Children’s Hospital and the AGCT core at Cedars-Sinai Cancer.

### IF staining

As described above, sections No. 2 and 4, and 1 and 5 were 0 µm, and 10 µm, apart from the GE section, respectively. Section No. 2 was sequentially stained with P2RY12/GFAP/AT8, section No. 4 with Olig2/Tau46/Aβ, section No. 1 with WFS1/AT8, and section No. 5 with GAD1. IF staining was performed as previously described [14]. All sections were air dried at 60 °C for 10 min and then fixed and permeabilized by prechilled acetone at −20 °C for 15 min. For sections No. 2 and 4, slides were immersed in 1 × PBS for 3 h at 37 °C for antigen retrieval. For sections No. 1 and 5, antigen retrieval was performed by incubating slides in 10 mM sodium citrate (pH6.0, 95 °C) for 12 min. Antibodies were incubated sequentially to avoid false-positive results coming from co-immunostaining. On day 1, after 1-h blocking by 10% donkey serum (in 1 × PBS), P2RY12 (1:1000) and Olig2 (1:500) primary antibodies were applied on section No. 2 and 4, respectively, overnight at 4 °C, while GAD1 (1:100 in PBS with 10% donkey serum) antibody was applied to sections No. 1 and 5 overnight at 4 °C. On day 2, after three washes with 1 × PBS, secondary antibodies were incubated with corresponding sections for 2 h at 37 °C. The sequential staining was followed by 1-h re-blocking with 10% donkey serum in 0.3% PBST, primary antibody combinations for each section were incubated at 4 °C in the same way as described above for regular IF staining. The nuclei were stained with Hoechst33342. Autofluorescence was quenched with 0.5 × TrueBlack solution in 70% ethanol for 10 min. The coverslips were mounted with Fluoromount-G Mounting Medium, and the slides were then imaged by Zeiss Axio Observer microscope.

### H&E and IF staining image alignment

The 20 × tile images taken by Zeiss Axio Observer microscope were exported into merged and individual channel images by Zeiss Zen software (2.6 blue edition). Since adjacent continuous sections were collected from the same brain tissue block, the shape outline and the structure of each section are identical (Additional file 1: Fig. S1A). The merged channel images were landmarked according to DAPI and hematoxylin staining in corresponding H&E images using ImageJ “Multi-points” tool (Additional file 1: Fig. S1A). Multi-points landmarks in each merged channel image were then saved and applied to individual channel images. Image alignment was performed using the “Transform/Landmark correspondences” plugin in ImageJ as described by other groups [16]. After the transformation, IF staining of AT8 and Aβ, as well as other staining like Tau 46 were used to check whether the alignment was accurate. H&E image and transformed channel images were stacked into.tiff image and imported into Loupe Browser (v5.0.1) and Space Ranger (v1.2.2) for further alignment with Visium spots.

### Annotation of cortical layers and the WM of the human MTG

To assign Visium spots to their corresponding layers, we first combined all 25,293 spots from our six samples and generated a cross-sample Uniform Manifold Approximation and Projection (UMAP) via Seurat (v.4.0.5) [28] pipeline to visualize dimension-reduced data in 2D space with unsupervised manner. All spots were clustered into eight groups and visualized in the original sample using eight colors assigned to each cluster. Although UMAP can roughly reflect the cortical layers’ information and separate the WM from the gray matter well, it is not powerful to segregate six layers in the gray matter of cortex. Since certain layer(s) may be missing during the brain dissection or mounting brain sections on the GE slide, arbitrarily assigning clusters from Seurat clusters into a sample may not be accurate without manual annotations. Moreover, the pathology development in AD brains may also change the gene expression profile and introduce more confounders in clustering. To solve this problem, we used both UMAP and IF staining as references to manually label each cortical layer and the WM.

In order to assign Visium spots to corresponding layers, H&E, IF staining, and previously identified layer-specific marker genes were used to define each layer. Previously identified layer-specific markers for seven layers were downloaded from the publicly available reference [29]. We first identified Layer I, Layer II, and the WM using GFAP, WFS1, and Olig2 staining from adjacent sections (Additional file 1: Fig. S2). Layer IV, the internal granular layer with a denser nuclei distribution between Layer II and the WM [30], can be defined using H&E staining on the GE slide. PCP4 gene expression was visualized on the spatial map to show up Layer V [29]. Layer III was defined as the layer between Layer II and Layer IV, and Layer VI was defined as the layer between Layer V and the WM. Spots with tissue loss or folded regions were dropped as the noise. All the spots across six samples were allocated to a certain layer or assigned as the noise based on the UMAP, H&E, and/or IF staining. A total of 245 spots were dropped as the noise across six samples.

### Selection of AD pathology-associated spots and adjacent spots.

After the alignment of all 11 channels, the signal from each channel was adjusted to clearly present the staining. Visium spots with AD pathology (Aβ^+^, AT8^+^, and Aβ^+^/AT8^+^) in AD cases were selected based on Aβ, and AT8 staining. By checking the spots with Aβ staining, we noticed that there are three different types of plaques in our Visium samples: compact plaques, cored plaques and diffused plaques. In order to include all three different types of Aβ plaques and exclude the staining without the characteristics of plaques, we randomly chose the spots with Aβ staining, and measured the minimum size (i.e., diameter = 10 μm, as previously reported [16] of compact plaques and cored plaques, and the minimum number (i.e., n = 20) of small plaques within diffused plaques. We used those two values as a cutoff and manually measured the size and number of each Aβ staining within Loupe Browser to define the Aβ + spots. Using the same strategy, we randomly chose the spots with AT8 staining and measured the minimum size (i.e., diameter/length = 15 μm) of NFTs and the minimum number (i.e., n = 5) of short neuropil threads within a spot. In this way, we identified sufficient Aβ + , AT8 + , Aβ−, and AT8- spots for downstream analysis.

Before the selection of AD pathology-associated spots, the contrast for all images was adjusted to 50% in Loupe browser to show a clear edge of Aβ or AT8 staining. Aβ^+^ spots were defined as those spots which cover either a single big plaque (diameter ≥ 10 µm) or multiple small plaques (diameter < 10 µm and plaque number ≥ 20). Similarly, AT8^+^ spots were those covering single intraneuronal neurofibrillary tangles (diameter/length ≥ 15 µm) or multiple neuropil threads (length ≥ 5 μm and the number over 5). Aβ^+^/AT8^+^ spots were defined as those overlapping spots between Aβ^+^ and AT8^+^ spots. After each pathology-associated spot was labeled, the immediately surrounding spots without AD pathology were labeled as level 1 spots, spots without AD pathology surrounding level 1 were labeled as level 2 spots, and the spots without AD pathology surrounding level 2 spots as level 3 spots. The selected spots labeled as noise in the manual annotation step were automatically dropped and would not be included in the following data analysis.

### FASTQ generation, alignment, and quantification

Raw sequencing data, named Binary Base Call files (BCL), of six human MTG samples were generated from the Illumina NovaSeq6000 sequencer. BCL files were converted to FASTQ files by using “mkfastq” function from SpaceRanger (v.1.2.2) software toolkit. FASTQ files were then aligned to and quantified to the expression matrix by using GRCh38 Reference-2020-A and the SpaceRanger “count” function. The two functions SpaceRanger “mkfastq” and “count” were used for demultiplexing sample and transcriptome alignment using default parameters.

### Data processing

Seurat (v.4.0.5) was used for the following analysis. For a sample, the spatial file and HDF5 file obtained from SpaceRanger were loaded into Seurat by the function “Load10X_spatial”. Then, we used the recommended sctransform method [31] to normalize counts by the function “SCTransform” in its default settings. Multiple manual annotations (meta information) were added to each Seurat object via the function “AddMetaData.” All the spots labeled as noise were removed, and the remaining spots were used for the following analyses.

### Data Integration, dimension reduction, spot clustering, and visualization

The Seurat integration framework was used for identifying clusters and removing batch effect from the six samples (three AD and three CT cases). First, 3,000 features (Additional file 3: Table S2) were selected for integrating six datasets via setting the nfeatures parameter as 3,000 via the function “SelectIntegrationFeatures.” Then the functions “PrepSCTIntegration” and “FindIntegrationAnchors” were used for preparing anchor features and generating anchors for the following analyses. Principal Component Analysis (PCA) was utilized for dimension reduction and denoising via “runPCA.” The top ten principal components (PCs) were selected to perform UMAP via “runUMAP” in the default settings. Spot clustering results were generated by the functions “FindNeighbors” and “FindClusters,” and the UMAP plots and spatial maps were generated by “DimPlot” and “SpatialPlot.”

### Visualize the layer-specific genes on spatial maps

Layer-specific genes identified in this study were regarded as individual gene modules, i.e. Layer 1 module: *SPARC*, *CXCL14*; Layer 2 module: *HPCAL1*, *CALB2*; Layer 4 module: *RORB*, *NUAK1*; Layer 5 module: *PCP4*, *SMYD2*, *SNCA*, *RAB3C*, *SLC25A22*; Layer 6 module: *MAP2K1*, *DIRAS2*, *KRT17*; White Matter module: *MBP*, *CLDND1*, *BCAS1*, *MTURN*, *PAQR6*, *HIPK2*, *DYNC1LI2*. There are no Layer 3-specific genes conserved in all six samples. The gene module score was calculated by using the function “AddModuleScore” by default parameters to indicate their average expression. Then, we visualized the classification capability for MTG architecture of each gene module using the ggplot2 R package, in which the gene module score in each spot was highlighted by colors. Similarly, the classification capability for MTG architecture of each individual gene was also visualized via spatial maps by using the ggplot2 R package, in which colors denoted the expression of each gene in spots.

### Validation of identified layer-specific markers on public datasets

Four public datasets were used for validating and visualizing layer-specific gene modules identified in this study. Expression matrices and spatial information of two samples (Sample ID: Adult Human Brain 1 and Adult Human Brain 2) were downloaded from https://www.10xgenomics.com/products/spatial-gene-expression. Expression matrices and spatial information of two samples (Sample ID: 151673, 151674) from Maynard’s dataset [29] were downloaded from Globus data transfer platform via the accessible endpoint “jhpce#HumanPilot10x” for http://research.libd.org/globus/. Gene module score was calculated as described in our *Visualize the layer-specific genes on spatial maps* section.

### WGCNA analyses of highly variable genes in six samples

The R package WGCNA (v.1.70.3) [32] was used to build co-expression networks and identify gene modules, which include negative and positive correlations. The top 10,000 highly variable genes [16] in each sample were selected from the “vst” assay using “FindVariableFeatures” in Seurat v.4.0.5. The six sets of selected highly variable genes were merged using the “unique” function, and their expression profiles were merged using the “merge” function in Seurat v.4.0.5. Soft power 3 was chosen by the WGCNA function “pickSoftThreshold”. Then, the function “TOMsimilarityFromExpr” was used to calculate the TOM similarity matrix via setting power as 3, networkType as unsigned, and TOMType as unsigned. The distance matrix was generated by substrating the values from similarity adjacency matrix by one. The function flashClust (v.1.01) [33] was used to cluster genes based on the distance matrix, and the function “cutreeDynamic” was utilized to identify eight gene modules by setting deepSplit as 3. To determine key genes with the highest degree, the function “TOMsimilartyFromExpr” was used to generate the similarity matrix based on all genes of each module. The matrix was then used for calculating the edges’ closeness centrality score via function “graph_from_adjacency_matrix” and “closeness” from the R package igraph (v.1.2.6) for eight modules. Based on the closeness score, genes were sorted, and the top-30 genes were selected to generate four modules circos plot via the circlize package (v.0.4.13) [34].

### Significance test for gene modules

To validate the biological significance of the eight gene modules derived from the WGCNA analysis, we perform enrichment analysis between identified eight gene modules and 14 AD-associated gene lists in the public domains, respectively. The R package GeneOverlap (v1.26.0) was used to perform Fisher’s exact test, resulting in odds ratio, number of overlaps, and *p*-values. Then, the Benjamini–Hochberg method was applied to adjust the *p*-values. The enrichment results with an adjusted *p*-value $\le 0.05$ were retained and considered as statistically significant enrichment.

### DEG and GO enrichment analysis

The DEG analysis was conducted by the Seurat function “FindMarkers” via grouping AD and CT cases with default parameters. The DEGs were selected if the adjusted *p*-value was less than 0.05. Based on the identified DEGs, the enrichment analyses of GO terms (Biological Process) were performed via the R package clusterProfiler (v.3.18.0) [35] using the functions of “enrichGO”. The enrichment analysis results were filtered out if the adjusted *p*-value was greater than 0.05. The sidebar legend was colored by the gene ratio defined as hit genes over the query genes via “enrichGO” function. For each identified gene module, the component genes were used for pathway enrichment analysis.

To determine the layer-specific marker genes in both AD and CT samples, the six samples saved in Seurat Objects were firstly merged using the “merge” function in Seurat v.4.0.5, and then layer-specific markers conserved across the six samples based on the layer annotation were predicted using the function “FindConservedMarkers” [36] in Seurat v.4.0.5 under default parameters.

### Jaccard/Tanimoto similarity test

To confirm whether the sample size is sufficient for DEG analyses of all layers, we performed Jaccard/Tanimoto similarity test on the layer-specific markers conserved across subsampled subsets of the six samples. The Jaccard/Tanimoto similarity test on the six subset levels was described below.

Subset level 1 (1/6): we randomly selected one from the six samples using exhaustion method, leading to six subsets. Each subset was used to compute the conserved layer-specific markers.

Subset level 2 (2/6): we randomly chose two from six samples using exhaustion method, resulting in ${C}_{6}^{2}=15$ subsets. Each subset was applied to calculate the conserved layer-specific markers.

Subset level 3 (3/6): we randomly picked three from six samples using exhaustion method, resulting in ${C}_{6}^{3}=20$ subsets. Each subset was utilized to obtain the conserved layer-specific markers.

Subset level 4 (4/6): we randomly selected four from six samples using exhaustion method, resulting in ${C}_{6}^{4}=15$ subsets. Each subset was used to obtain the conserved layer-specific markers.

Subset level 5 (5/6): we randomly chose five from six samples using exhaustion method, resulting in ${C}_{6}^{5}=6$ subsets. Each subset was applied to obtain the conserved layer-specific markers.

Subset level 6 (6/6): we picked all the six samples and obtained ${C}_{6}^{6}=1$ subset. This subset was utilized to obtain the conserved layer-specific markers across all the samples.

Jaccard index of each subset at the different levels was computed via the formula below.

$${\mathrm{Jaccard}}_{i,j}=\frac{B\cap {S}_{i,j}}{B\cup {S}_{i,j}}$$

where set B was defined as the all-conserved layer-specific markers predicted from the six samples; set S was defined as conserved markers calculated from different subset strategies; index $i$ indicated subset levels (from level 1 to level 6); index $j$ indicated the number of the subset in the $i$ ^th^ level.

Note that since Sample AD-2 does not contain Layer I, no layer-specific marker was identified from this layer. Besides, no layer-specific markers of Layer III were identified as conserved markers in six samples.

### RNAscope HiPlex assay in combination with post-IF staining of Aβ/AT8/GFAP

In order to validate and quantitate the gene expression changes around AD pathology-related regions (Aβ^+^, AT8^+^, and Aβ^+^/AT8^+^), we performed the smFISH assay using the RNAscope HiPlex12 Ancillary Kit to simultaneously detect 18 different RNA targets selected from DEGs associated with Aβ plaques and/or AT8^+^-tangles/neuropil threads. The process was performed according to the manufacturer’s user manual with a few modifications. Fresh frozen sections were fixed in 4% PFA at room temperature for 1 h. The sections were washed twice by 1 × PBS and dehydrated sequentially in 50%, 70% and 100% ethanol for 5 min. After permeabilization by protease IV, the sections were incubated with the premixed probes in the ACD HybEZ II oven for 2 h at 40 °C, and three amplification steps in 40 °C were extended to 45 min. HiPlex Fluoro Tail mixture was incubated with the sections for 25 min in 40 °C after amplification steps. After each round of HiPlex Fluoro incubation, the nuclei were counterstained with DAPI for 30 s, and the lipofuscin autofluorescence was quenched by 1 × True Black for 5 min. The slides were mounted with Fluoromount-G Mounting Medium, and 40 × Tile image were taken by Zeiss Axio Observer microscope after each round. Before the next round, signals were quenched using the cleaving buffer and washed twice with 1 × PBS containing 0.5% of Tween20. Post-IF staining of AT8/Aβ/GFAP was performed after RNAscope HiPlex12 assay as previously described.

Since regions captured by imaging from a sample were the same from each round of RNAscope and post staining, DAPI staining did not change much after each round’s processing. Tile images from each round were stitched and aligned to each other according to DAPI channel using ImageJ. The same nuclei from matched rounds were identified by “Extract SIFT Correspondences” plugins in ImageJ. After the multi-points labels of each DAPI were given and saved, those labels would be applied to all the other channels from a same round and be transformed to the template image (DAPI in round 1 in all cases) using “Landmark Correspondences” plugins in ImageJ. All the transformed images were then overlayed and merged using Zeiss Zen microscope software (2.6 blue edition). The density of DAPI and the GFAP staining was used as reference for annotate each cortical layers and the WM.

The same regions of interests were extracted from Layer II/III and layer V of each sample for the following quantification. Merged images were imported into ImageJ to assign cell types to each cell. Each cell was manually circled according to DAPI and assigned a cell type according to their corresponding *RBFOX3* (neurons), *P2RY12/C1QB* (microglia), *MBP* (oligodendrocytes), and *GFAP* (astrocytes) RNA probes. Cells expressing none of those cell type marker genes were discarded in the following analysis. Circled cells from a same cell type were added to ROI manager and saved for the quantification of RNAscope puncta. The particle size cutoff for RNAscope puncta was set as 0.15 µm^2^ to 2.5 µm^2^ in Analyze Particles in ImageJ based on the average size of the RNAscope puncta. ROIs from one cell type were applied to different channels and analyzed using Analyze Particle command in Macro in ImageJ. The number of RNA counts from different channels were matched to their corresponding RNA probes and assigned to the same cell in that ROI for further analysis.

To perform the proximity to pathology analysis, we first generated “pathology-masks” of Aβ or AT8 staining in AD samples used to define the ROIs defined in the last step (Additional file 1: Fig. S3). Aβ and AT8 staining were imported into ImageJ separately and converted into binary masks as Aβ or AT8 “pathology-masks” (Additional file 1: Fig. S3A). In order to remove the potential false staining, masks with sizes smaller than 35 µm^2^ in Aβ, and 5 µm^2^ in AT8 were excluded from the following analysis. The remaining masks were expanded by 55 µm (average diameter of Aβ plaques) from the edge of Aβ masks, or 10 µm (average diameter of AT8^+^ neurons) from the edge of AT8 masks to generate “proximal to pathology” masks (Additional file 1: Fig. S3B). “Pathology-masks” were further extended 165 µm from the mask edges in Aβ, or 30 µm from the mask edges in AT8 to generate “distal to pathology” masks (Additional file 1: Fig. S3C). ROIs from the same sample were applied to these two sets of masks, and the median intensity was measured within each ROI. The median intensity was used to define a consistent population of cells across samples that were either in “proximal to pathology” or “distal to pathology”. “Proximal to pathology” cells were defined as cells covered by “proximal to pathology” masks, whose median intensity is 255 (white parts in Additional file 1: Fig. S3B), while “distal to pathology” cells were defined as those cells uncovered by “distal to pathology” masks, whose median intensity is 0 (black parts in Additional file 1: Fig. S3C). Aβ/AT8 double positive regions were selected by overlapping Aβ and AT8 staining masks. Aβ/AT8 double positive masks smaller than 5 µm^2^ were dropped. The “proximal to pathology” masks and “distal to pathology” masks were generated in the same way as described above. For each probe, the counts of RNA puncta in ROIs from the same cell type were compared within layer II/III or layer V between three AD and three CT samples, or between “proximal to pathology” cells and “distal to pathology” cells using nonparametric Mann–Whitney test in Prism 5.

### Spatial integration of snRNA-Seq and SRT data from human MTG

The snRNA-Seq dataset (n = 15,928 total nuclei passed the quality control) from the human MTG [37] was used for performing spatial transcriptomic deconvolution analysis for our six samples. This MTG dataset was downloaded at http://celltypes.brain-map.org/. There were 15,603 nuclei annotated with 75 cell subpopulations in this dataset, and we combined all the subpopulations from the same cell type to give seven broad cell types for the following deconvolution.

The Cell2location performs deconvolution in two steps, i.e., training regression model and building cell2location model. In the train regression model part, the function “filter_genes” was used to filter the genes based on default parameters. Then, the function “cell2location.models.RegressionModel.setup_anndata” by default parameters was used for building an annData object. After applying the function “RegressionModel” to create a regression model, the function “mod.train” under parameters “max_epochs = 250, batch_size = 2500, 'num_samples' = 1000, use_gpu = True” was used to train this regression model. Based on the trained model, functions “adata_scrna_raw. varm” and “adata_scrna_raw.var” were implemented to export estimated expression in each cell subpopulation. Functions “np.intersect1d”, “adata_vis”, and “inf_aver.loc” was used to find shared genes and subset both the annData object and reference signatures. For the model construction part, function “cell2location.models.Cell2location.setup_anndata” was used to prepare the anndata for constructing the cell2location model. Functions “cell2location.models.Cell2location” and “mod.train” was used for to create and train this model.

### Statistical analysis

No statistical methods were used to predetermine sample sizes, since (*i*) a rigorous statistical framework for design of SRT experiments is missing in the literature and (*ii*) existing tools for the design of single-cell genomic experiments are not appropriate for SRT experiments and do not consider key factors of SRT experiments. In our study, each brain sample has over 4,000 spots and ~ 3500 genes per spot, which provides sufficient power for DEG analysis of Visium SRT datasets. Prism 5 software was used to analyze the data. All data are expressed as mean ± SEM. We performed the D’Agostino–Pearson omnibus normality test to determine whether the data were normally distributed or the *F* test to determine whether the data assumed equal variances. We then chose the following statistical tests. Nonparametric Mann–Whitney tests were used to compare the number of single RNA dots in each cell human CT and AD. All Pearson correlation coefficients were computed by Prism 9.2.0. All results represent two-sided tests comparing groups of biological replicates. *P* < 0.05 was considered statistically significant for all measures. The *n* values represent the number of spots or cells in each group; exact values are indicated in figure legends.

## Results

### SRT in human postmortem MTG from AD and CT cases

**Fig. 1:**
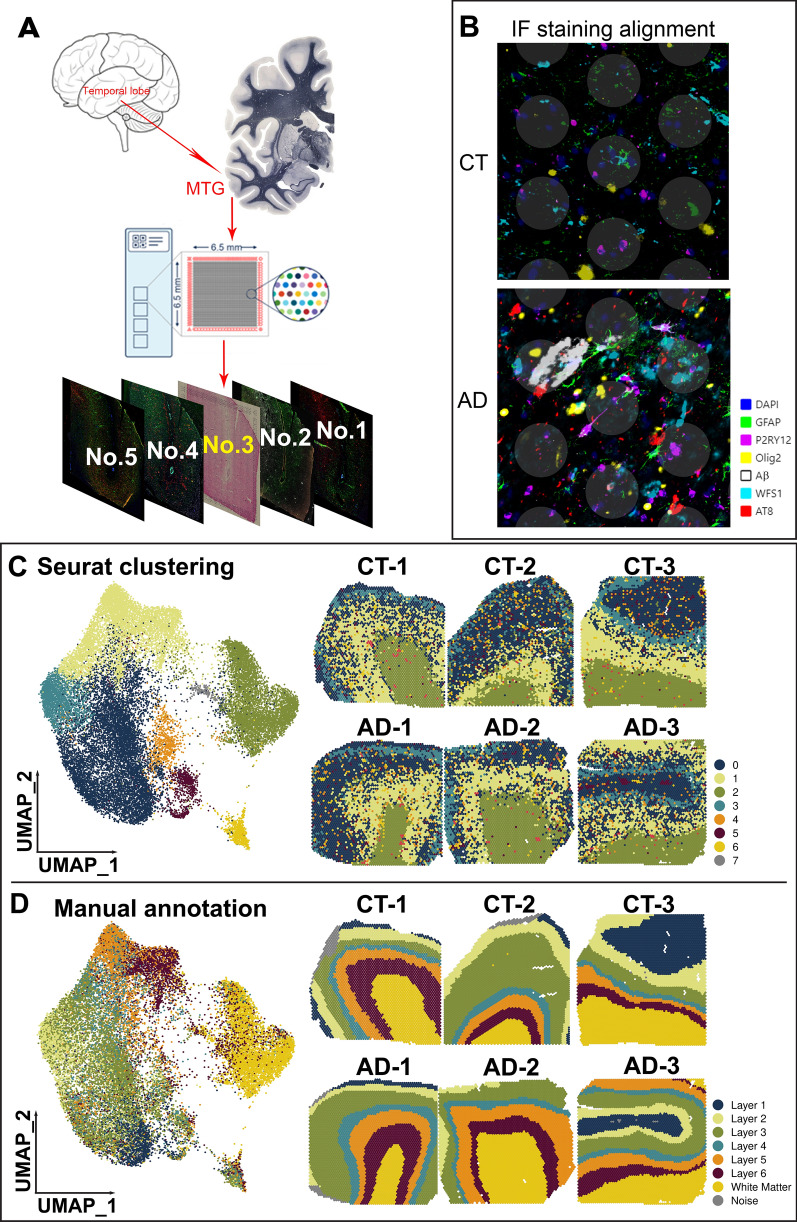
Spatial transcriptomics (SRT) of the human middle temporal gyrus (MTG). (**A**) Sequential 10 µm sections of control (CT) and Alzheimer (AD) MTG brain regions were used for all experiments. The middle section (No. 3) was used for SRT on the Visium platform, and the four adjacent sections (No.1, 2, 4, 5) were used for immunofluorescence (IF) staining. (**B**) Aligned image of SRT with IF staining of nuclei (DAPI, blue) and cell-type specific markers (GFAP (green), P2RY12 (purple), Olig2 (yellow), WFS1 (teal)) and AD pathological hallmarks (Aβ plaques (white) and AT8 + pathological tau (red)). (**C**) Uniform manifold approximation and projection (UMAP) plots show eight clusters (0–7) were identified by the Seurat integration framework using Visium spots from both CT (CT-1, CT-2 and CT-3) and AD (AD-1, AD-2 and AD-3) human MTG. The right panel shows spatial maps of the eight clusters for each individual sample generated from Seurat clustering. (**D**) Manual annotation of six cortical layers and the adjacent white matter (WM). The left panel figure shows the manually labeled spots (six cortical layers and the WM) on UMAP space based on the Seurat integration framework. The right panel of the spatial maps show the localization of the manually labeled spots for each individual sample. Noise, spots with tissue loss or folded regions

Human postmortem fresh frozen brain sections from the MTG of three CT (Braak stages I-II, male, 58, 72 and 82 years old) and three AD (Braak III-IV, male, 86, 86 and 89 years old) cases (see detailed case demographics, clinical and neuropathological information in Additional file 2: Table S1) were chosen to perform the 10 × Genomics Visium experiments. Due to the limited availability of brain tissues with good RNA integrity and the gender difference in AD pathology, we only chose males for this study. The potential biological replicates (i.e., sample size) and covariates (e.g., age, post-mortem interval, RNA integrity, tissue intactness and ApoE genotype) that may affect gene expression results are also acknowledged in the Discussion. For each sample, five continuous Sects. (10 µm) were saved and labeled sequentially as No. 1 to No. 5. The middle section (No. 3) was mounted on the Visium Gene Expression (GE) slide for SRT profiling. Sections (No. 1, 2, 4, 5) were used for immunofluorescence (IF) staining of cell-type markers and AD pathology (Fig. 1A). We obtained a total of 25,293 Visium spots from all six samples, and we detected 8,377 ± 1,482 unique molecular identifiers (UMIs) and 3,395 ± 419 unique genes per Visium spot (Additional file 3: Table S2, and https://bmbls.bmi.osumc.edu/scread/stofad-2). Two hundred and forty-five spots with tissue loss or folded tissue were classified as noise and not used for further analysis.

Given that all the adjacent sections are within 20 µm from the GE section (No. 3) and the average cell-body size of most nerve cells in the human brain is ~ 10–20 µm, the cell distribution and brain architecture can be used to align adjacent sections to the GE Sect. [16]. Therefore, we aligned the GE section with the hematoxylin and eosin (H&E) staining and four adjacent sections with IF staining to annotate the cortical layers, the WM and AD pathology-associated regions. The No. 1 section was co-stained with WFS1/AT8 (pS202/T205 tau), No. 2 with P2RY12/GFAP/AT8, No. 4 with Olig2/Tau46/Aβ, and No. 5 with GAD1 antibodies (Fig. 1A, B, and https://bmbls.bmi.osumc.edu/scread/stofad-2). GFAP (astrocytes) and Olig2 (oligodendrocytes) were highly expressed in layer I [38–40] and the WM [41, 42], respectively. WFS1 (wolframin) was used as a layer II marker [29]. AT8 and Aβ antibodies were used to stain pathological tau and Aβ plaques, respectively. AT8^+^ tau was enriched in layers-II and V of human AD. Tau46 antibodies were used to stain all the neurons expressing tau protein in both CT and AD cases. DAPI (nuclei dye) from each section was used as references to align all antibody staining to the GE section (Additional file 1: Fig. S1A). Then Visium spots with AD-associated pathology (Aβ^+^, AT8^+^, and Aβ^+^/AT8^+^) were chosen to identify gene signatures associated with these pathological changes in AD samples (Fig. 1B and https://bmbls.bmi.osumc.edu/scread/stofad-2). Although the alignment of Visium spots with AD-associated pathology from an adjacent section may not fully capture the AD-associated pathology from a same cell directly on the GE section, we still can use this alignment to predict the regions with spots that would cover regions of Aβ and/or tau pathology on the GE section (Additional file 1: Fig. S1D). The rationale of this prediction is based on the thickness of the section, the size of Aβ plaques and the specific enrichment of tau pathology in layers II and V in human AD at Braak stages III-IV. Our IF staining of AT8 on adjacent sections showed very similar localizations (Additional file 1: Fig. S1D), further demonstrating the feasibility of using adjacent sections to predict AD-associated spots on the GE section.

### Defining layer-specific gene expression and architecture of the human MTG

The laminar architecture of the human cortex is closely related to its function and vulnerability to different pathology [43, 44]. For example, neurons in layers II, III and V of the temporal lobe including the MTG and the entorhinal cortex are particularly vulnerable to tau pathology in human early AD [7, 8, 11, 45, 46]. Therefore, to identify gene signatures specific to each layer and figure out how they are differentially expressed in disease, we first assign Visium spots to their corresponding layers in each sample, then we generated a uniform manifold approximation and projection (UMAP) of pooled six samples and spatial map of each sample with Seurat clusters (Fig. 1C). Although UMAP can assign a unique cluster to the WM, it cannot distinguish six layers in the gray matter of the cortex into individual clusters in the spatial map. In contrast, manual annotation of layers based on H&E staining and IF staining of layer-specific markers (see details in the Methods) can clearly define six cortical layers, unless missing, and the WM (Fig. 1D).

**Fig. 2:**
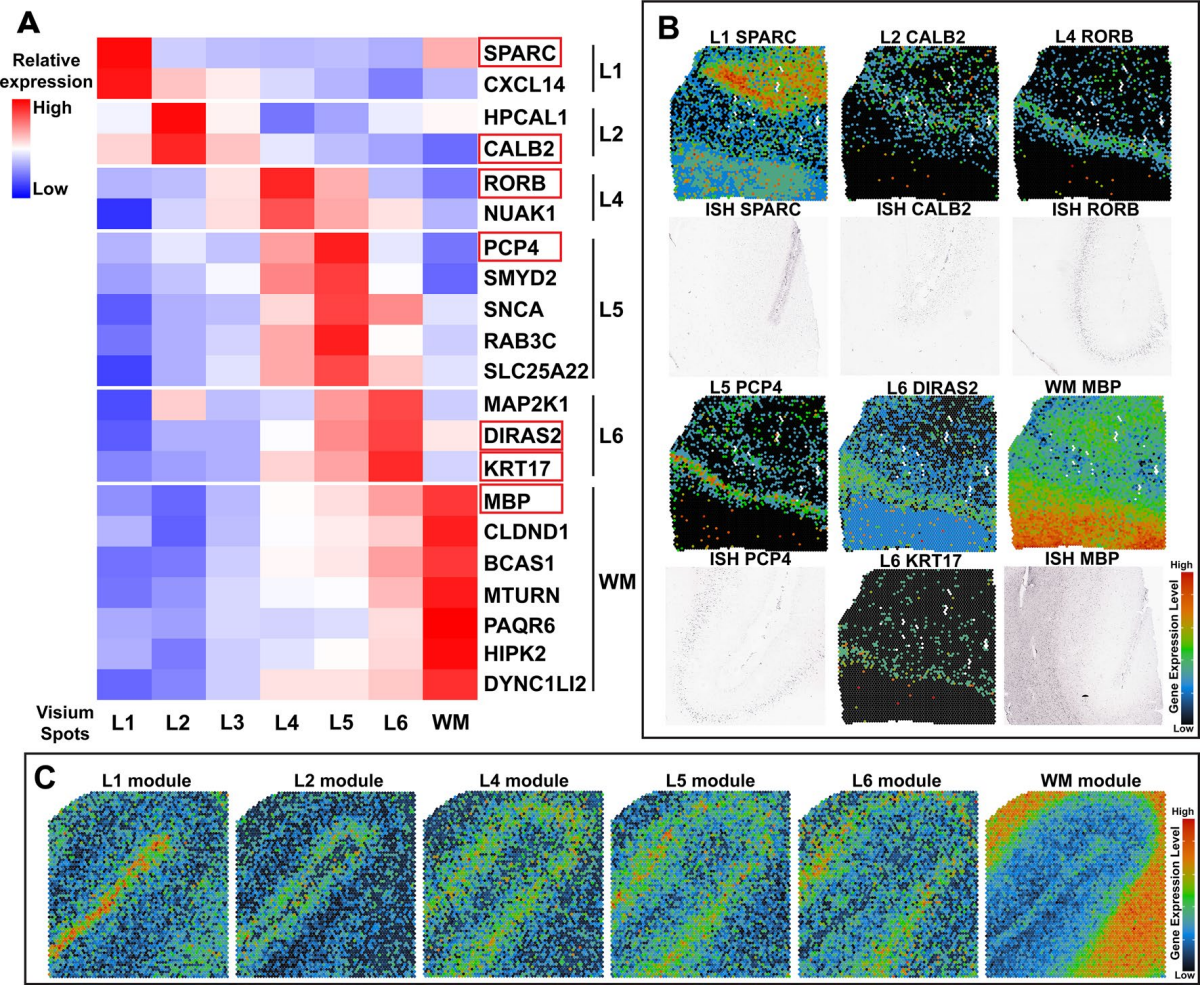
Layer-specific genes define the anatomical architecture of the human MTG and the frontal cortex. (**A**) Heatmap of Z-scores for layer-specific marker genes identified from CT and AD human MTG. The heatmap shows the expression levels of layer-specific marker genes in each layer except layer III, which does not show any conserved marker genes in all 6 samples. The red color indicates a relevantly higher expression than other layers, while the blue color indicates a lower expression. Identified layer-specific marker genes, SPARC (L1: layer I), CALB2 (L2: layer II), RORB (L4: layer IV), PCP4 (L5: layer V), DIRAS2 and KRT17 (L6: layer VI), and MBP (the WM), are boxed in red and were used for validation of cortical laminae in spatial maps using Loupe Browser shown in (**B**). (**B**) Spatial maps of layer-specific marker genes in our samples (color images) were validated by in situ hybridization (ISH) data (gray images) from the Allen Brain Institute’s Human Brain Atlas (Sample ID: 79,205,802, 80,225,788). ISH data is not available for DIRAS2 and KRT17. (**C**) Public available 10 × Visium SRT data of the human frontal cortex (Sample ID: Human Brian 1) is annotated by our layer-specific marker gene modules

Based on manual annotation of each layer, Visium spots were assigned to their corresponding layer for each sample. To identify layer-specific marker genes that are applicable to both AD and CT cases, layer-specific Visium spots from all six samples were combined as a cluster and underwent DEG analysis against Visium spots in other layers, excluding noise spots. We analyzed the datasets by pooling all six samples together using the Seurat package to define layer-specific marker genes (Fig. 2A). Since the layer III-specific marker gene (HOPX) was conserved across only five samples, we did not include it in the heatmap (Fig. 2A). In order to further demonstrate whether six samples can identify conserved layer markers, we performed the Jaccard/Tanimoto similarity test [47] for six samples. The results (Additional file 1: Fig. S4) showed that when the sample size exceeds five, the conserved marker genes were very consistent no matter which sample combination we chose. Therefore, a sample size of six will have sufficient power to identify conserved marker genes. The layer-specific marker genes (Fig. 2A and Additional file 4: Table S3) were then visualized on spatial maps in Loupe Browser (Fig. 2B). Furthermore, the layer-specific expression of several marker genes was further validated by their in situ hybridization (ISH) data (Sample ID: 79,205,802, 80,225,788, Temporal cortex) from the Allen Brain Institute’s Human Brain Atlas (Fig. 2B). To demonstrate that those layer-specific marker genes would not be affected by AD condition, we generated the heatmap separately using layer-specific marker genes in CT or AD (Additional file 1: Fig. S5A), which shows a similar result to the heatmap in Fig. 2A. To verify if the identified layer-specific marker genes can be used to define the laminar architecture of human frontal cortex in publicly available datasets, we calculated the average expression value of layer-specific marker genes on Visium SRT data (Sample ID: Human Brain 1 and Human Brain 2) generated by 10 × Genomics (Fig. 2C and Additional file 1: Fig. S5B) and another group [29] (Sample ID: 151673, Additional file 1: Fig. S5B and https://bmbls.bmi.osumc.edu/scread/stofad-2). Overall, we identified not only layer-specific markers shown by other studies [29, 37, 48] (e.g., RORB, PCP4, MBP) but also novel marker genes (e.g., SPARC, CALB2, DIRAS2, KRT17) that have not been reported. We also demonstrate that the layer-specific marker genes we identified are powerful in defining the anatomical architecture of the human MTG and the frontal cortex.

### Prediction of cell type proportions in cortical layers and the WM of human MTG

**Fig. 3:**
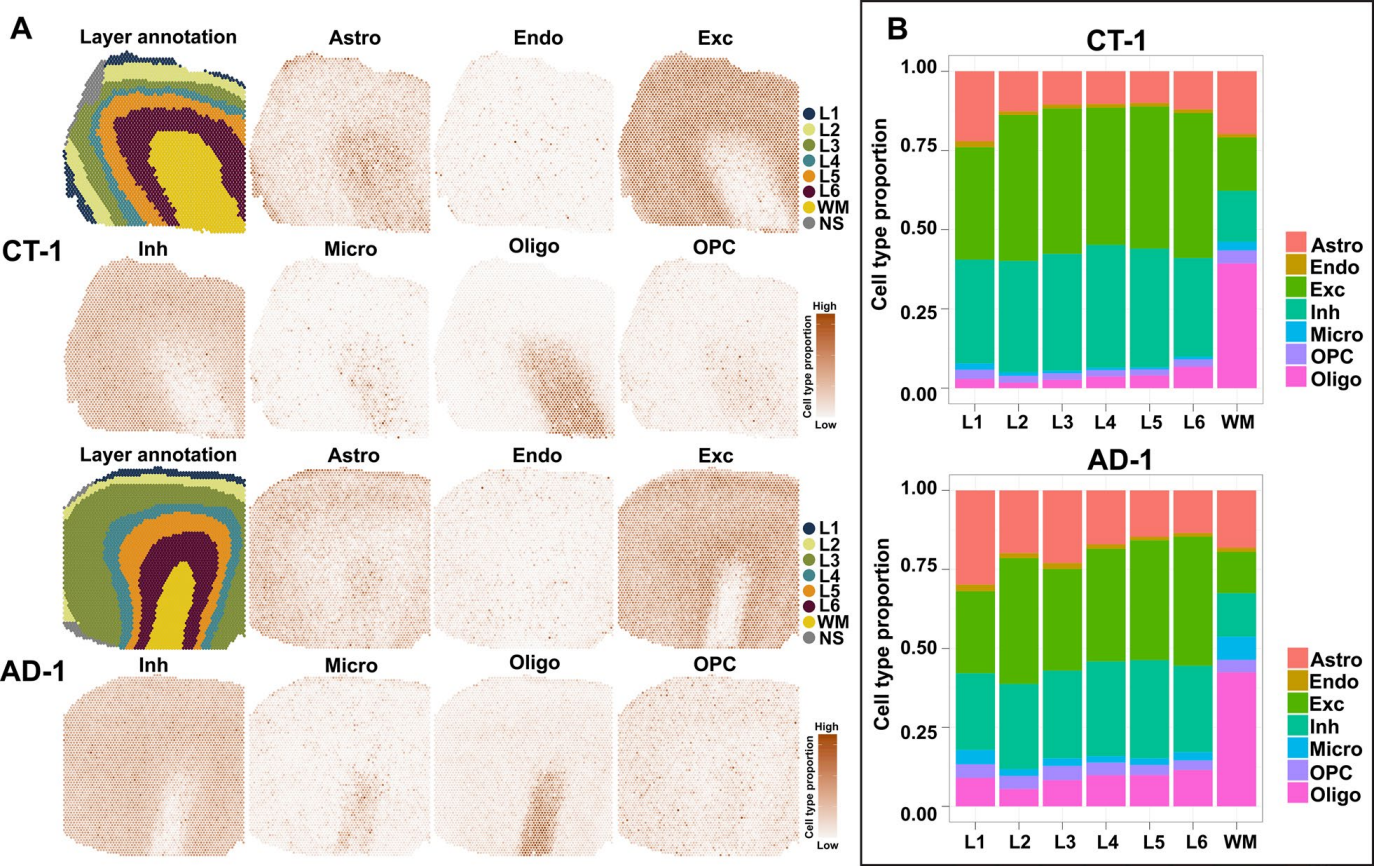
Cell type deconvolution analysis of snRNA-seq data and Visium SRT data from human MTG. (**A**) Manual annotations of six cortical layers and the adjacent WM in CT (CT-1) and AD (AD-1) human MTG, and spatial maps of the cell-type-specific distributions of seven main cell types (Astro, Endo, Exc, Inh, Micro, OPC and Oligo) [37] among spots and layers corresponding to these two cases using Cell2location (see details in Methods). Spots enriched for each cell subpopulation are shown in brown, which is divided into high and low expression based on the proportion of each individual cell subpopulation. (**B**) Stack bar plots highlight the proportion of seven main cell types in layers I-VI (L1-L6) and the adjacent WM of CT-1 and AD-1. NS, noise spots; Astro, astrocytes; Endo, endothelial cells; Exc, excitatory neurons; Inh, inhibitory neurons; Micro, microglia; Oligo, oligodendrocytes; and OPC, oligodendrocyte precursor cells

Since 10 × Visium spots cannot reach the single cell level, one way of predicting cell type distributions is to integrate the snRNA-seq and the Visium data for conducting cell type deconvolution analysis [49, 50]. To investigate the cell type distribution in each cortical layer and the WM, we performed such an analysis using the best performance tool (Cell2location) [50, 51] for our six samples with publicly available snRNA-Seq datasets of the human MTG [37]. The results of Cell2location analysis [51] showed that excitatory (Exc) neurons are predicted to be mainly located at layers II-VI (Fig. 3, Additional file 1: Fig. S6, and Additional file 5: Table S4), which is consistent with the selective vulnerability of excitatory neurons in AD [11, 12, 29] and the major distribution of Aβ plaques and neurofibrillary tangles (NFTs, AT8^+^) in such layers [7, 8, 10, 46, 52]. This analysis also revealed a higher percentage of astrocytes in layer I and oligodendrocytes in the WM than in other layers in human MTG (Fig. 3, Additional file 1: Fig. S6, and Additional file 5: Table S4). To explore whether early-stage AD pathology affects the proportion of different cell types, we compared the proportion of each cell type between CT and AD cases in each cortical layer and the WM (Additional file 1: Fig. S6B). The cell type proportions did not significantly change in each cortical layer and the WM of AD, except microglia which significantly increased (log2 fold change = 1.336, *p*-value = 0.007) in the WM of AD compared to CT (Additional file 1: Fig. S6B).

To compare our predicted cell type proportions in each cortical layer from our deconvolution analysis with previously published MTG cell type proportions [53] in corresponding layers, we calculated the Pearson correlation coefficients (Additional file 5: Table S4) for proportions between our prediction and the reference across all cell types for each layer. Our predicted proportions of astrocytes, endothelial cells, excitatory neurons, and oligodendrocytes show a significant correlation with the cell type proportion result using probabilistic cell typing by in situ sequencing (pciSeq) [53] (astrocytes: r = 0.997, *p*-value = 0.0008; endothelial cells: r = 0.893, *p*-value = 0.0083; excitatory neurons: r = 0.883, *p*-value = 0.0098; oligodendrocytes: r = 0.800, *p*-value = 0.028). The low correlation of inhibitory neurons (r = 0.518, *p*-value = 0.1462), microglia (r = −0.305, *p*-value = 0.278), and oligodendrocyte precursor cells (r = 0.579, *p*-value = 0.114) might be due to the absence of the WM information in the pciSeq dataset and the difference in resolution/sequencing depth of two SRT methods.

To determine if AD pathology is mainly found in excitatory neuron-vulnerable layers of AD cases, we quantitated the number of spots with AD pathology (Aβ^+^, AT8^+^, and Aβ^+^/AT8^+^) and normalized to the total number of spots in corresponding cortical layers and the WM. Generally, it was found that the normalized proportion of spots with AD pathology is higher in layers II-V than in other layers and the WM (Additional file 6: Table S5), although three samples for each layer are not sufficient to perform statistical tests. This is consistent with the predicted high proportions of excitatory neurons in layers II-VI and the selective excitatory neuronal vulnerability of these layers in AD [11, 43, 46, 54].

### DEG analysis between AD and CT cases

**Fig. 4:**
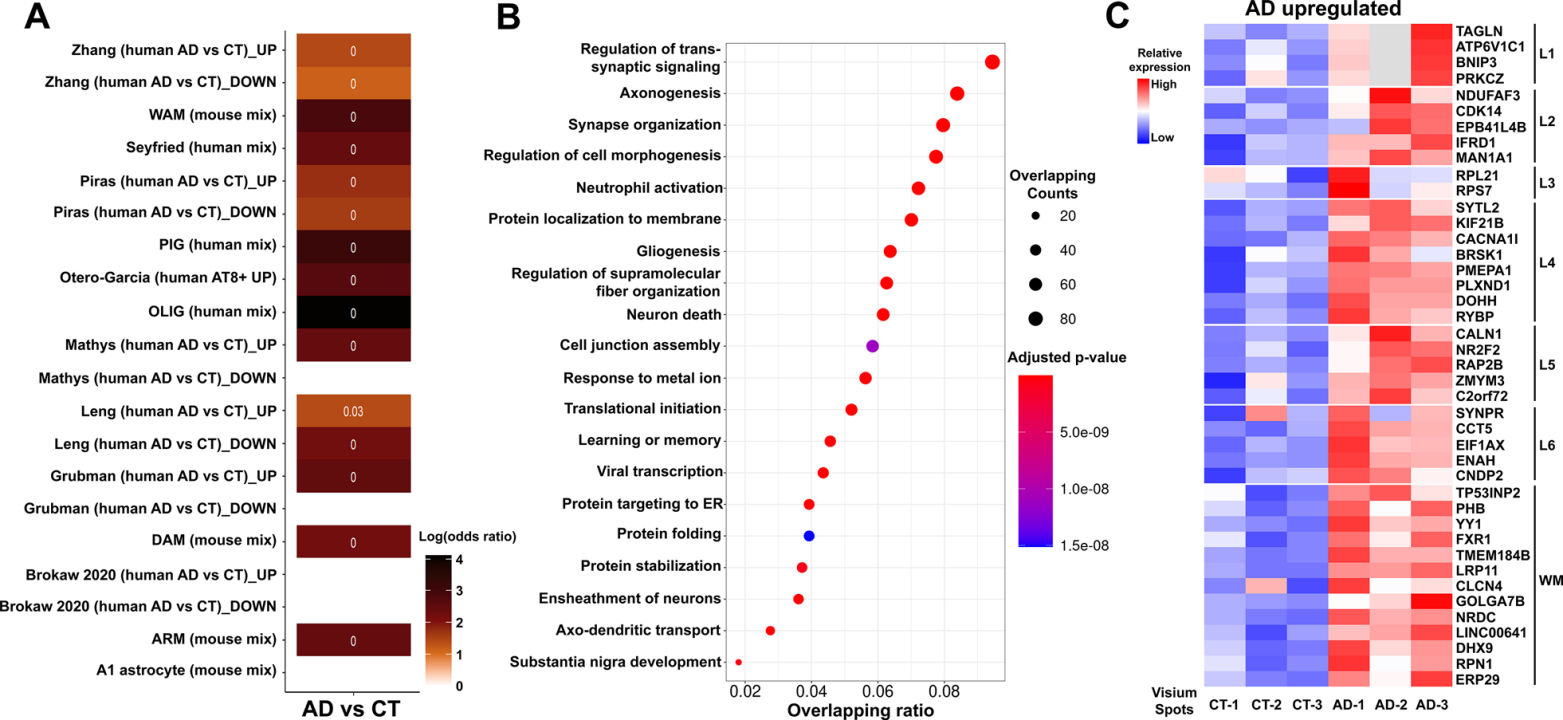
Differentially expressed genes (DEGs), Top-20 enriched pathways and layer-specific DEGs in AD vs CT human MTG. (**A**) The Fisher’s exact test between DEGs (AD *vs* CT 1008 upregulated) identified in our study and 14 previously published AD-related transcriptomic and proteomics datasets (Additional file 7: Table S6). The x-axis and y-axis indicate objects of Fisher’s exact test. Heatmap block color indicates the log-transformed odds ratio. Each heatmap block is labeled by its adjusted *p*-value. If the heatmap block has a non-significant adjusted *p*-value, the block color was assigned as white. WAM, WM-associated microglia; PIG, plaque-induced gene; OLIG, oligodendrocyte gene; DAM, disease-associated microglia; ARM, amyloid-response microglia; A1, A1 astrocytes. (**B**) Gene Ontology (GO) pathway analysis of DEGs between AD and CT cases. DEGs were assessed with the Seurat FindMarkers function with a log-fold-change threshold of 0.25. Bonferroni-adjusted *p*-values were used to determine significance at an FDR < 0.05. Dot plot shows the top 20 enriched GO terms which represent biological process terms from the DEGs between AD and CT. Each dot is colored by the Benjamini–Hochberg adjusted *p*-value. The dot size is scaled by the number of overlapping genes with the related GO terms. The x-axis is scaled by the ratio between the overlapping count and the total number of genes of the term. Gene set enrichment analysis was performed using the Enrichr web server. (**C**) Heatmaps of Z-scores for layer-specific upregulated DEGs identified between AD and CT cases. The heatmaps show the expression levels of layer-specific DEGs in each layer for each AD and CT case. Layer I of AD-2 is shown in gray since this layer was not present in our sampled brain block. The red color indicates a relevantly higher expression than other cases, while the blue color indicates a lower expression

To better understand how early AD pathology affects the global gene expression in the human MTG, we performed DEG analysis by combining the Visium spots in three AD *vs* three CT samples. We compared the gene expression between AD and CT samples and found 1,008 genes were significantly upregulated, while 14 were downregulated in AD samples (Additional file 7: Table S6). Interestingly, some DEGs have also been identified in recently published Microarray [20], bulk RNA-Seq [21, 22], snRNA-Seq [12, 13, 15], single-soma RNA-Seq [55], single microglia RNA-Seq [24] and proteomic datasets [25] from human AD and CT cases. The gene set enrichment analysis (Fishers' exact test) between DEGs (AD *vs* CT upregulated, 1008 in total) and 14 previously published AD-related gene lists showed significant overlaps (Fig. 4A and Additional file 7: Table S6). Furthermore, the Gene Ontology (GO) enrichment analysis of these upregulated DEGs reveals many AD-related biological processes such as regulation of trans-synaptic signaling, axonogenesis, neutrophil activation involved in immune response, gliogenesis, protein targeting to membrane and ER, regulation of supramolecular fiber organization, neuron death, response to metal ion, viral transcription, protein folding, and ensheathment of neurons, etc. (Fig. 4B and Additional file 7: Table S6). Since there are only 14 downregulated DEGs, we directly listed them in Additional file 7: Table S6 without performing the GO enrichment analysis of them.

Next, we identified the AD upregulated DEGs in layers I to VI and the WM (Additional file 8: Table S7). We further compared those DEGs and found layer-specific upregulated DEGs in AD cases (Fig. 4C and Additional file 8: Table S7), although there are shared DEGs among different cortical layers and the WM. These results suggest that differential gene expression exists in each cortical layer of the MTG, which may contribute to the regional vulnerability in early AD.

### Identification of co-expression gene modules associated with AD pathology

**Fig. 5:**
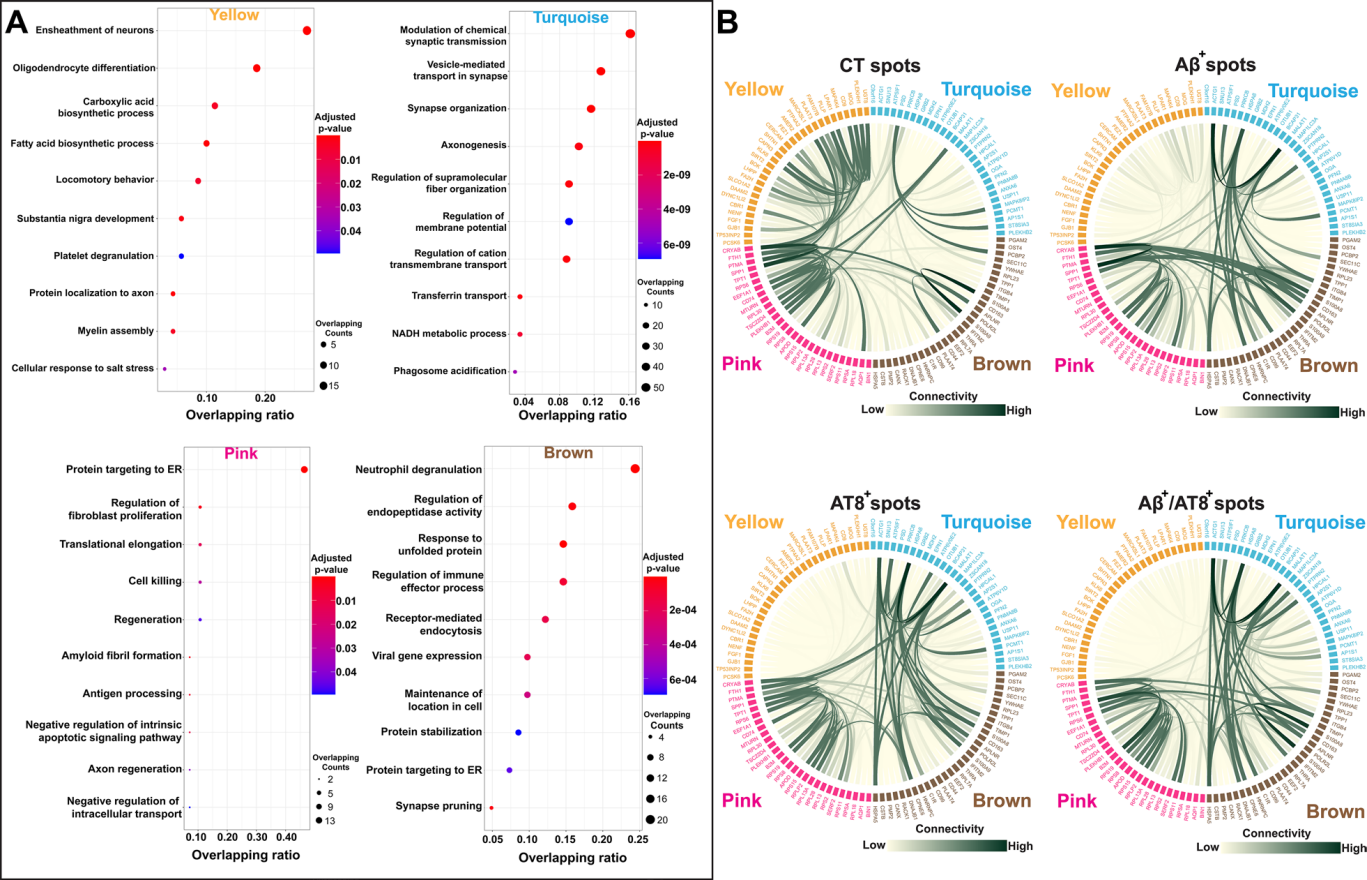
AD-associated gene modules and their co-expression patterns within areas of AD pathology. (**A**) Gene-ontology (GO) pathway analysis of four modules (Yellow, Turquoise, Pink and Brown) identified by weighted gene co-expression network analysis (WGCNA) of 10,000 highly variable genes from six samples (Additional file 1: Fig. S5). Dot plot shows top 10 enriched GO terms: biological process terms from the DEGs within each module (Additional file 9: Table S8). Each dot is colored by the Benjamini–Hochberg adjusted *p*-value. The dot size is scaled by the number of overlapping genes with the related GO terms. The x-axis is scaled by the ratio between the overlapping count and the total number of genes of the term. Gene set enrichment analysis was performed using the Enrichr web server. (**B**) Circos plots of the connectivity strength within and across modules for Visium spots in CT human MTG and Visium spots in AD human MTG with specific AD pathology (Aβ^+^, AT8^+^, and Aβ/AT8^+^). Circos plots are composed of four different modules (Yellow, Turquoise, Pink and Brown; top 30 hub genes from each module based on their closeness centrality score); nodes represent highly deregulated genes from each module; and edges (green lines) indicate connectivity (high: dark green, low: light green)

Despite many DEGs associated with Visium spots showing AD pathology have been identified in previous studies and our present study, it remains to be investigated how these DEGs are connected functionally at a system level. To this end, we performed weighted gene co-expression network analysis (WGCNA) [19] on 10,000 highly variable genes from six samples and identified eight gene modules in which genes with similar expression profiles and functions are grouped (Additional file 1: Fig. S7A and Additional file 9: Table S8). Furthermore, the GO enrichment analysis of the component genes in each module reveals that the Yellow module is enriched with biological processes like ensheathment of neurons, oligodendrocyte differentiation, carboxylic acid biosynthetic process, and fatty acid biosynthetic process; Turquoise module with synaptic transmission, synapse organization, axonogenesis and regulation of supramolecular fiber organization; Pink module with protein targeting to ER, translation elongation, cell killing and amyloid fibril formation; Brown module with neutrophil degranulation, regulation of endopeptidase activity, response to unfolded protein and regulation of immune effector process (Fig. 5A and Additional file 9: Table S8). It is noteworthy that there are significant overlaps between these eight gene modules and 14 published AD-related gene lists [12–15, 20–22, 24, 25, 55] identified previously in human AD *vs* CT cases, supporting the important roles of our newly defined gene modules in regulating the pathogenesis of AD (Additional file 1: Fig. S7B and Additional file 9: Table S8).

Based on the MA (log ratio versus abundance) plot (Additional file 1: Fig. S7A) of eight gene modules [56] and enrichment result with 14 datasets (Additional file 1: Fig. S7A and Additional file 9: Table S8), we focused on four modules (Yellow, Turquoise, Pink, and Brown) for downstream analysis. In order to investigate if and how AD pathology affects the co-expression of the four gene modules, we performed WGCNA of Top 30 hub genes selected from each module (120 genes in total) with the highest closeness centrality score using only Visium spots colocalized with positive staining for either one of three different AD pathologies (Aβ^+^, AT8^+^, or Aβ^+^/AT8^+^) from AD samples. All Visium spots from the CT cases served as CT spots. Our WGCNA analysis yields connectivity matrixes demonstrating the correlation patterns among those module-specific genes in response to different AD pathology. The results, visualized by the circos plots [57] showed that the co-expression gene relations within the Yellow module and between the Yellow and Pink modules in CT spots decreased in the presence of AD pathology, whereas the co-expression gene relations within Turquoise and Brown modules and between Turquoise, Brown and Pink modules markedly increased (Fig. 5b). Furthermore, the co-expression gene relations of these four modules changed as the distance (45–245 µm) from Visium spots with AD pathology increased (level 1: 45 µm; level 2: 145 µm; level 3: 245 µm) (Additional file 1: Fig. S8C, Additional file 10: Table S9, and https://bmbls.bmi.osumc.edu/scread/stofad-2) (see details in Methods for the definition of AD pathology-associated spots and 3-levels of spots). These results indicate the gene co-expression pattern is in a spatial and AD-pathology dependent manner.

### Identification of gene signatures and pathways associated with AD pathology

**Fig. 6:**
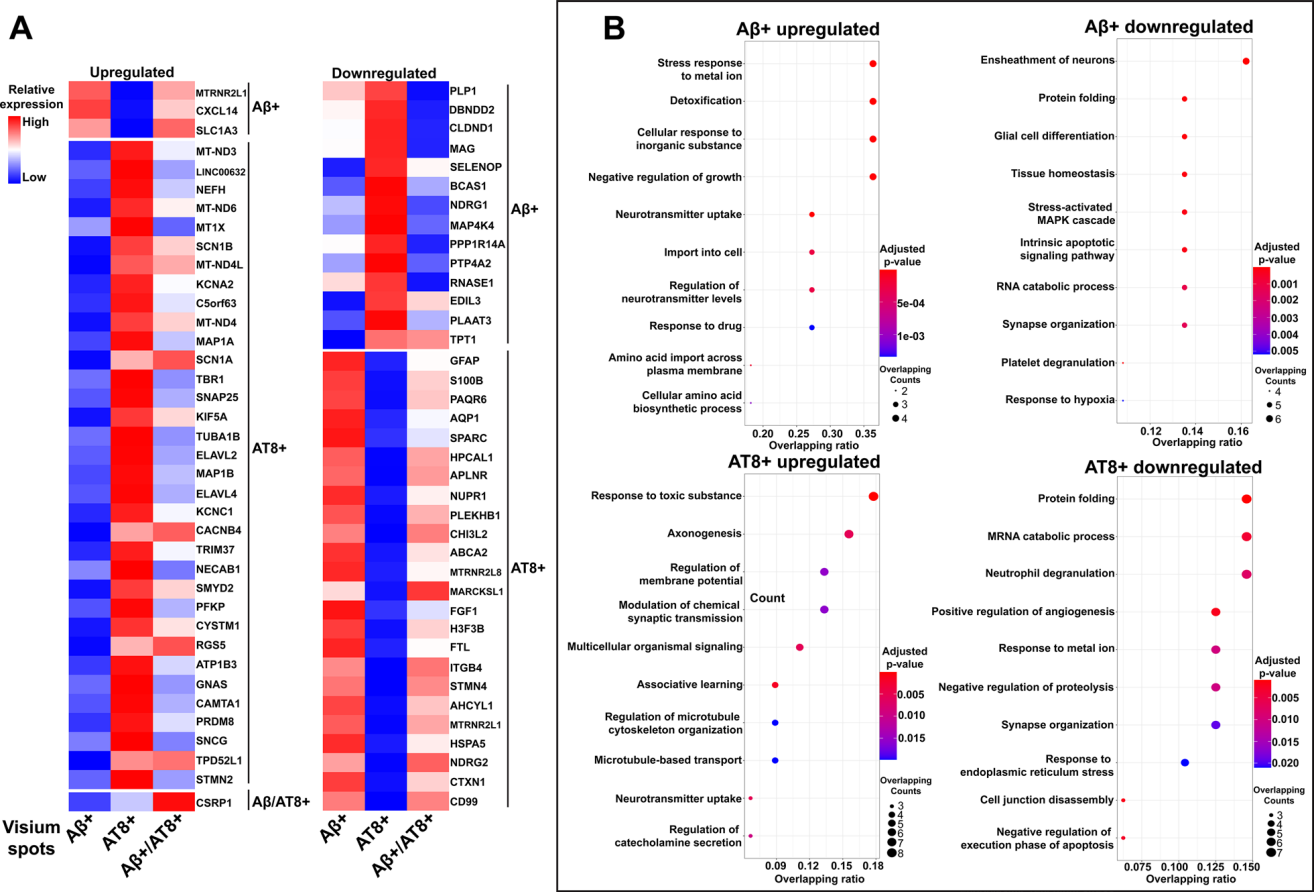
AD pathology-associated gene signatures and pathways. (**A**) Heatmap of Z-scores for upregulated and downregulated DEGs specific to Visium spots localized with different AD pathologies (Aβ^+^, AT8^+^, and Aβ/AT8^+^). DEGs were identified from pathological regions (either Aβ^+^, AT8^+^, and Aβ/AT8^+^) vs. surrounding level 3 spots of three AD samples at the pseudo-bulk level. The red color indicates a relevantly higher expression than other pathologies, while the blue color indicates a lower expression. (**B**) Gene ontology (GO) pathway analysis of identified DEGs (Additional file 10: Table S9) specific to Visium spots localized with Aβ plaques (top row) or pathological tau, AT8 (bottom row). The Dot plot shows top 10 enriched GO terms. Each dot is colored by the Benjamini–Hochberg adjusted *p*-value. The dot size is scaled by the number of overlapping genes with the related GO terms. The x-axis is the ratio and indicating the proportion of overlapping genes between the query gene list and all genes in the GO term

To further identify unique gene signatures and pathways associated with Visium spots showing AD pathology (Aβ^+^, AT8^+^, or Aβ^+^/AT8^+^), we performed DEG analysis between Visium spots with each AD pathology and surrounding level 3 spots (245 µm from the site of pathology). Compared to levels 1 and 2, level 3 spots are at a greater distance from the site of pathology and should exhibit a greater difference in gene expression. We identified many known and novel gene signatures associated with each AD pathology (Additional file 10: Table S9). Interestingly, we also found each AD pathology is related to unique DEGs (Additional file 1: Fig. S8). The upregulated and downregulated genes exclusively associated with each AD pathology were shown in Fig. 6A.

Next, we determined the biological processes that are enriched in each AD pathology via the GO enrichment analysis. Although there are shared pathways between different AD pathologies, each pathology does have unique upregulated and downregulated pathways (Fig. 6A and Additional file 10: Table S9). These results suggest that Aβ^+^-plaques and AT8^+^-tau pathology may induce unique and differential alterations of biological processes, at least in the early stages of AD, although Aβ is believed to be the upstream driver of tau pathology in genetic forms of AD [58].

### Validation of known and novel DEGs associated with AD pathology at the single-cell level using smFISH

**Fig. 7:**
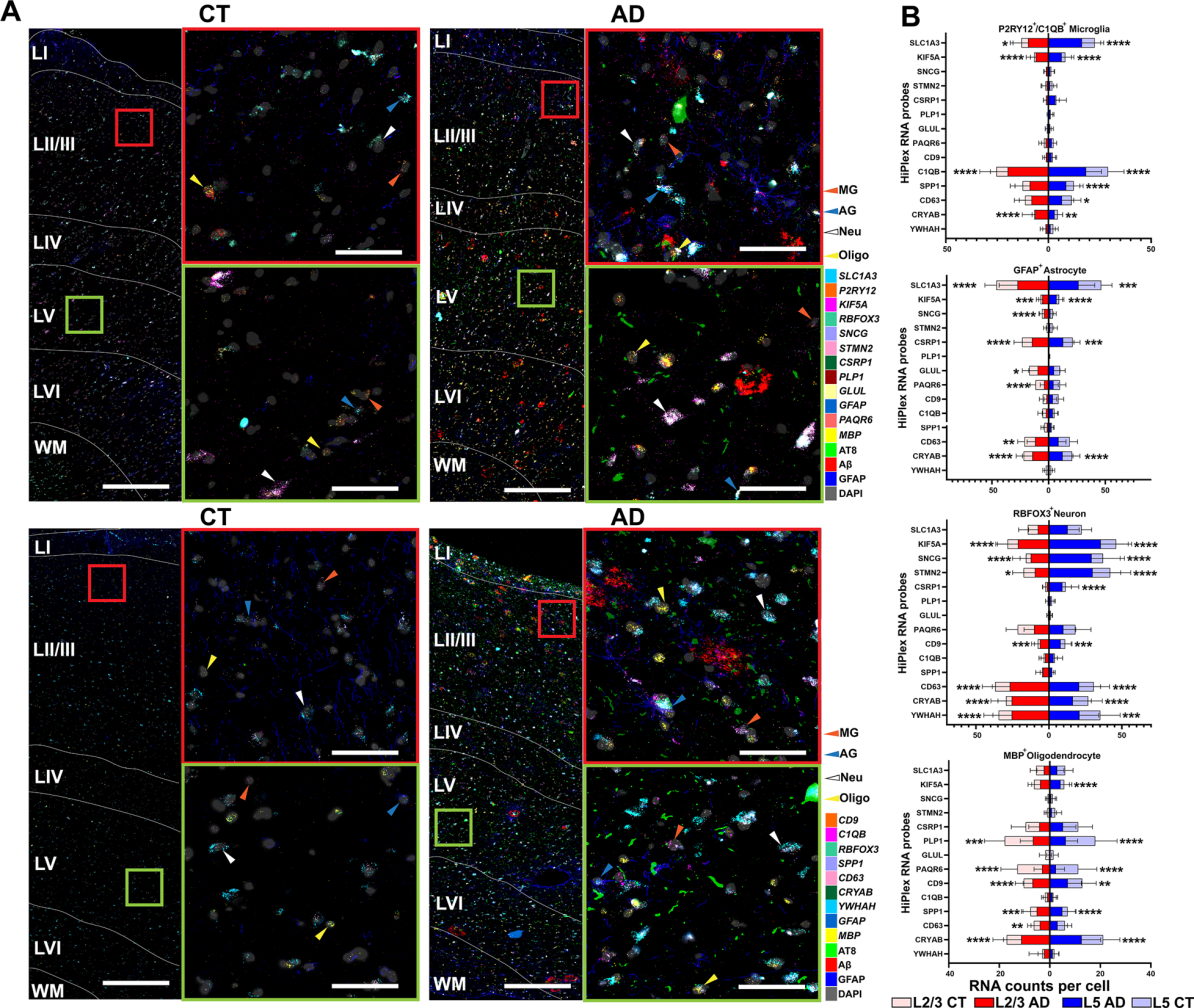
Validation of identified representative DEGs in layers II/III/V from AD and CT at the single-cell level using RNAscope smFISH. (**A**) Representative four rounds of whole-tiles RNAscope images were registered with their corresponding post-Immunofluorescence (IF) staining of Aβ, AT8, and GFAP (left panel of each quadrant). The representative high-mag images of layers II/III and V (left panel of each quadrant) were selected from the regions in the whole-tiles images circled by red (layer II/II) and green (layer V) rectangles. Individual cell was labeled as microglia (MG), astrocyte (AG), neuron (Neu), or oligodendrocyte (Oligo) according to its *P2RY12/C1QB*, *GFAP, RBFOX3,* and *MBP* expression respectively. (**B**) Stacked bar plots show the quantification of RNAscope probes against human *SLC1A3*, *KIF5A*, *SNCG*, *STMN2*, *CSRP1*, *PLP1*, *GLUL*, *PAQR6*, *CD9*, *C1QB*, *SPP1*, *CD63*, *CRYAB*, and *YWHAH* in microglia (*P2RY12*^+^*/C1QB*^+^), astrocytes (*GFAP*^+^), neurons (*RBFOX3*^+^), and oligodendrocytes (*MBP*^+^). RNA counts per cell were compared between three CT and three AD cases. For quantification, we identified 20–50 microglia, astrocytes, and oligodendrocytes, and 60–90 neurons from each case. Detailed information regarding quantification can be found in the methods section. * *P* < 0.05, ** *P* < 0.01, *** *P* < 0.001, **** *P* < 0.0001 (Mann–Whitney test, CT vs AD). Scale bar, 60 µm for layers II/II and V high-mag images, 360 µm for whole-tiles. See high-resolution RNAscope images at: https://bmbls.bmi.osumc.edu/scread/stofad-2

Since Visium does not generate single-cell level transcriptomics, we cannot infer the cell-type information of identified DEGs. To solve this issue, we chose RNAscope smFISH in combination with post-IF of Aβ plaques, NFTs and neuropil threads (AT8^+^), and GFAP to validate DEGs associated with these AD pathologies at the single-cell level using three AD (Braak III-IV) and three CT cases (Braak I-II). We selected 18 RNAscope probes against cell type-specific marker genes and AD pathology-associated DEGs identified in this study (Additional file 1: Fig. S9). Consistent with a previous report [59], *C1QB* and *SPP1* are mainly detected in microglia (*P2RY12*^+^/*C1QB*^+^), *CRYAB*, *CD9* and *PLP1* in oligodendrocytes (*MBP*^+^), *SLC1A3* and *GLUL* in astrocytes (*GFAP*^+^), *YWHAH* and *STMN2* in neurons (*RBFOX3*)^+^ (NeuN), and *CD63*, *KIF5A*, *SNCG* and *PAQR6* were detected in both neurons and glia, and *CSRP1* in astrocytes and oligodendrocytes (Fig. 7A and https://bmbls.bmi.osumc.edu/scread/stofad-2).

Given the vulnerability of layers II, III and V in human AD [7, 46], we focused on comparing the cell-type-specific RNA levels of above identified DEGs in those layers of MTG. Layer- and cell-type-specific analysis showed the significant increase of the RNA levels of *SLC1A3*, *KIF5A*, *C1QB* and *CRYAB* in microglia, *SLC1A3*, *KIF5A*, *CSRP1* and *CRYAB* in astrocytes, *KIF5A*, *SNCG*, *STMN2*, *CD9*, *CD63*, *CRYAB* and *YWHAH* in neurons, and *CD9*, *SPP1*, *CD63* and *CRYAB* in oligodendrocytes in both layer II/III and layer V in human AD compared to CT cases (Fig. 7B). Also, increased RNA levels of *SPP1* and *CD63* in layer V microglia, *SNCG*, *GLUL* and *CD63* in layer II/III astrocytes, *CSRP1* in layer V neurons, and *KIF5A* in layer V and *CD63* in layer II/III oligodendrocytes were found in human AD compared to CT cases (Fig. 7B). In addition, a significant decrease of the RNA levels of *PAQR6* in layer II/III astrocytes and in oligodendrocytes of layers II/III and V, and *PLP1* in oligodendrocytes of layers II/III and layer V was found in human AD compared to CT cases (Fig. 7B).

**Fig. 8:**
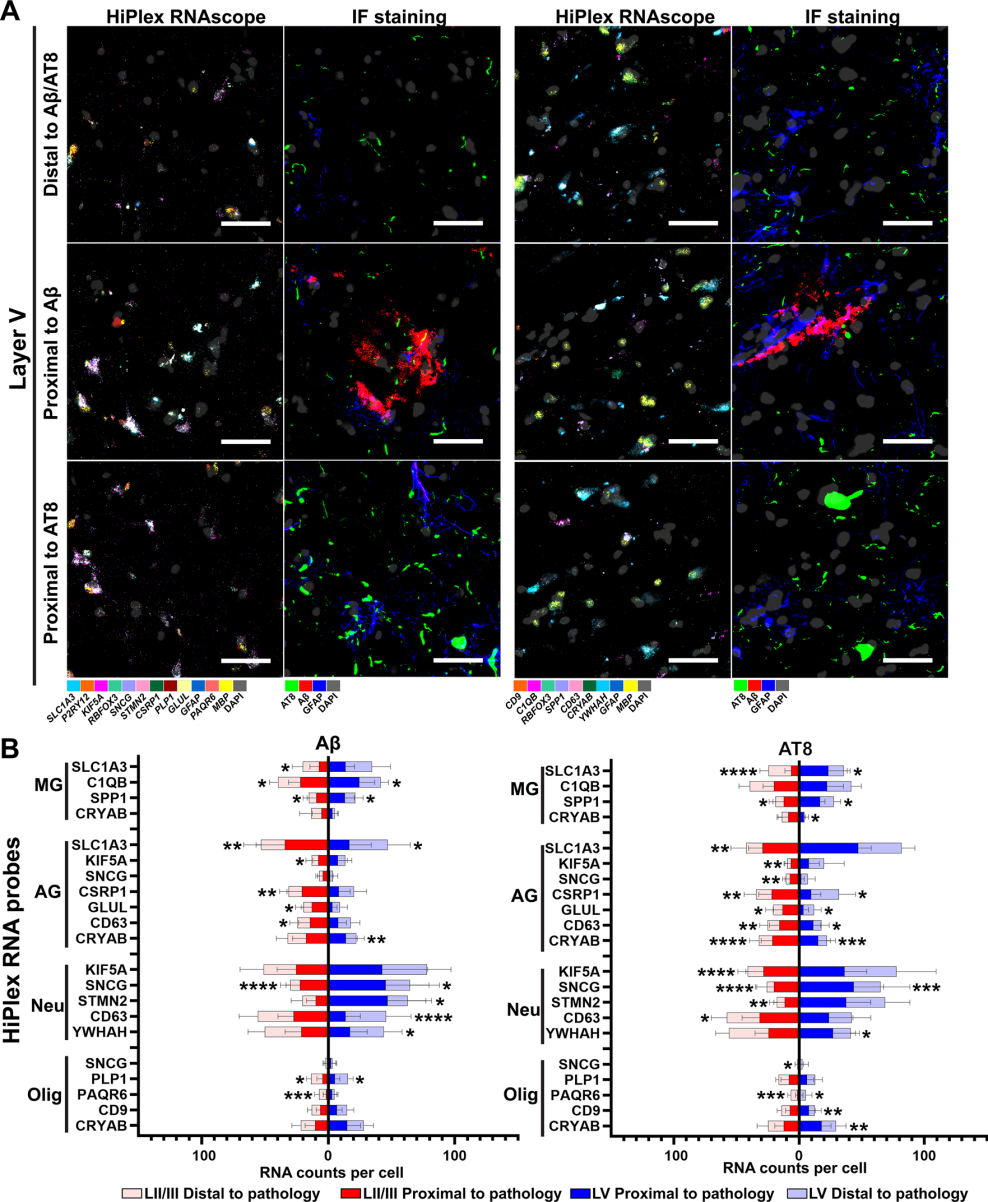
Validation of identified representative DEGs associated with AD pathology at the single-cell level using RNAscope smFISH. (**A**) Representative RNAscope images (left panel) and their corresponding post-IF staining of Aβ, AT8, and GFAP (right panels) in layer V for cells distal to and proximal to pathological spots. All images from the same cell were aligned and registered by imageJ (see details in Methods). (**B**) RNAscope probes against human *SLC1A3*, *KIF5A*, *SNCG*, *STMN2*, *CSRP1*, *PLP1*, *GLUL*, *PAQR6*, *CD9*, *C1QB*, *SPP1*, *CD63*, *CRYAB*, and *YWHAH* in microglia (*P2RY12*^+^*/C1QB*^+^), astrocytes (*GFAP*^+^), neurons (*RBFOX3*^+^), and oligodendrocytes (*MBP*^+^) were quantified and compared within three AD cases. For the quantification and definition of proximal to pathology/distal to pathology, please find the details in Methods. For quantification, we identified 20–50 microglia, astrocytes, and oligodendrocytes, and 60–90 neurons from each case. * *P* < 0.05, ** *P* < 0.01, *** *P* < 0.001, **** *P* < 0.0001 (Mann–Whitney test, in proximal to pathology vs distal to pathology). Scale bar, 50 µm. See high-resolution RNAscope images at: https://bmbls.bmi.osumc.edu/scread/stofad-2

Next, we investigated how closely those DEGs were associated with AD pathology such as Aβ plaques and/or NFTs and neuropil threads (AT8^+^). We found the RNA levels of *C1QB* and *SPP1* in microglia, *SNCG* in neurons of layers II/III and V were significantly increased proximal (55 µm) to Aβ plaques compared to those distal (> 165 µm) to Aβ plaques (Fig. 8 and Additional file 1: Fig. S10). Increased RNA levels of *SLC1A3*, *KIF5A*, *CSRP1*, *GLUL* and *CD63* in layer II/III astrocytes, *CRYAB* in layer V astrocytes, *STMN2* in layer V neurons; and decreased RNA levels of *SLC1A3* in layer II/III microglia and layer V astrocytes, *CD63* and *YWHAH* in layer V neurons, *PLP1* in layers II/III and V oligodendrocytes, and *PAQR6* in layer II/III oligodendrocytes were found proximal (55 µm) to Aβ plaques compared to those in the distal (> 165 µm) (Figs. 8 and Additional file 1: Fig. S10). We also found the increased RNA levels of *SPP1* in microglia, *CD63* and *CRYAB* in astrocytes, *SNCG* in neurons, and the decreased RNA level of *PAQR6* in oligodendrocytes of layers II/III and V proximal (10 µm) to NFTs and neuropil threads (AT8^+^) compared to those in the distal (> 30 µm) (Fig. 8 and Additional file 1: Fig. S10). We found the increased RNA levels of *SLC1A3* and *CRYAB* in layer V microglia, *SLC1A3*, *KIF5A*, *SNCG*, *CSRP1* and *GLUL* in layer II/III astrocytes, *KIF5A*, *STMN2* and *CD63* in layer II/III neurons, *YWHAH* in layer V neurons, *SNCG* in layer II/III oligodendrocytes, and *CD9* and *CRYAB* in layer V oligodendrocytes; and decreased *SLC1A3* in layer II/III microglia and *CSRP1* and *GLUL* in layer V astrocytes proximal (10 µm) to NFTs and neuropil threads (AT8^+^) compared to those in the distal (30 µm) (Fig. 8 and Additional file 1: Fig. S10). In addition, increased *CSRP1* and *GLUL* in astrocytes, *C1QB* and *SPP1* in microglia, and *SNCG* in neurons at layer II/III proximal to both Aβ plaques and NFTs and neuropil threads (AT8^+^) compared to those in the distal (> 165 µm) (Additional file 1: Fig. S10). Importantly, we are the first to identify three unique genes (e.g. *KIF5A*, *PAQR6* and *SLC1A3*) that are associated with AD pathology (Fig. 8 and Additional file 1: Fig. S10) using transcriptomic methods, besides those genes identified by other groups [13, 16, 24, 60–64]. We further investigated how those RNAscope results correlated with our Visium data. We compared the probe genes which were also found in 1008 AD upregulated DEGs with their corresponding Visium data. As shown in Additional file 1: Fig. S11, the fold change of average counts per cell of selected probes in AD highly correlated with their Visium gene expression fold change in AD (Pearson r = 0.80, *p*-value = 0.0002), which shows the reliability of DEGs identified using the SRT method.

## Discussion

Our study is among the first to implement Visium SRT technology in human AD brain tissue. We define the anatomical architecture of cortical laminae and the WM (Fig. 1D) and identify unique gene signatures (Figs. 2A, 4C, and 6A) and biological pathways (Figs. 4B and 6B) that may contribute to the vulnerability of various AD pathology. Furthermore, previously reported genes and novel genes identified in this study and their association with two main AD pathological markers (Aβ plaques and NFTs or neuropil threads) in layers II/III and V are confirmed at the single-cell level by RNAscope HiPlex smFISH assay, including *SLC1A3*, *KIF5A*, *SNCG*, *STMN2*, *CSRP1, PLP1*, *GLUL*, *PAQR6, CD9*, *C1QB*, *SPP1*, *CD63*, *CRYAB*, and *YWHAH* (Fig. 7 and Additional file 1: Fig. S10). To be noted, we did not include late AD cases at Braak stages V–VI in this study because we wanted to exclude the downstream gene changes that occur in late AD. We sought to evaluate the transcriptomic changes in the early phase of the disease because we are interested in defining biomarkers of early disease. Previous studies also indicate that major transcriptional changes appear early in AD [12, 13].

### Identification of novel layer-enriched marker genes in human MTG and reproducibility of these identified marker genes in human cortex with similar architecture

Cortical laminar-specific marker genes in human MTG have been previously identified by in situ hybridization of targeted ~ 1000 genes important for neural functions at a cellular resolution [48], and by snRNA-Seq at the single-cell level [37]. Recently, Visium in the adult human dorsolateral prefrontal cortex (DLPFC) [29] not only validated laminar enrichment of some canonical layer-specific genes that were previously identified in the rodent [65] and human cortex [37, 48], but also identified a number of previously underappreciated layer-enriched genes in human DLPFC [29]. Several genes identified as cell-type markers in specific cortical layers of human MTG are also enriched in the same cortical layers of human DLPFC [29, 48], suggesting laminar markers are conserved in human cortex with similar layer architecture.

By applying Visium in human MTG from both AD and CT cases, we identified conserved DEGs and gene clusters that correspond to anatomical layers of human MTG (Fig. 2A). Some cortical layer- and WM-specific marker genes were consistent with previous reports [29, 37, 48], including *RORB* (layer IV), *PCP4* (layer V), and *MBP* (WM). Importantly, we also identified novel marker genes for layers I-VI and the WM (Fig. 2A), which were validated by ISH data from the Allen Institute for Brain Science (Fig. 2B) and by annotating the specific cortical layers and the WM in publicly available Visium SRT data of the human frontal cortex (Fig. 2C). This again demonstrates the conservation of laminar markers in human MTG and frontal cortex. The identification of new layer-enriched marker genes will not only help us understand the anatomical architecture of the human cortex but also determine the layer-specific cellular vulnerability to AD pathology (e.g. the excitatory neuronal vulnerability in layers II-III of human temporal cortex [11, 12] and frontal cortex [29]). Unique marker genes identified in this study can be used for annotating the cortical layers and the WM in both healthy CT and AD cases (Additional file 1: Fig. S5A), which can avoid potential confounding effects of AD pathology on gene expression in corresponding layers of AD samples. With the aid of efficient annotation, we found that the proportion of spots with AD pathology which is normalized to the total number of spots in that layer is much higher in layers II-V compared to other layers (Additional file 6: Table S5), which suggests that these cortical layers may be vulnerable to AD pathology (Aβ plaques and NFTs). The cell type deconvolution analysis using Cell2location also predicted the higher proportion of excitatory (Exc.) neurons in layers II-VI, astrocytes in layer I, and oligodendrocytes in the WM compared to other layers (Fig. 3 and Additional file 1: Fig. S6). The predicted high proportions of excitatory neurons in layers II-VI may also explain the selective excitatory neuronal vulnerability of these layers in AD.

### Comparing the gene signatures, pathways and gene modules identified in this study with those identified by other techniques

We identified 1,022 (i.e., 1,008 upregulated and 14 downregulated) DEGs (Additional file 7: Table S6) that differentiate AD from CT cases. One potential reason of many more upregulated DEGs than downregulated DEGs might be the lack of significant neuronal loss in our AD cases at early Braak stages (Additional file 1: Fig. S6B). The mean expression RBFOX3 (NeuN) is significantly higher in AD than CT in our Visium Experiment, and the RBFOX3 RNA counts/cell also significantly increased in AD samples quantified by HiPlex RNAscope assay (Additional file 1: Fig. S11). Interestingly, those DEGs showed significant overlaps with previously published 14 AD-related datasets (Fig. 4A). The GO enrichment analysis of the 1,008 upregulated DEGs revealed biological process pathways (Additional file 7: Table S6) that may contribute to the development and progression of AD pathology. To be noted, Top-20 enriched pathways (Fig. 4B) have been identified in previous AD datasets [12, 13, 15–17]. We also identified the AD upregulated DEGs in layers I to VI and the WM (Additional file 8: Table S7). By removing the duplicate genes across all layers and WM, we identified the layer-specific DEGs upregulated in AD cases (Fig. 4C and Additional file 8: Table S7), which may shed a light on the regional vulnerability of AD in the future research.

Furthermore, WGCNA of 10,000 highly variable genes identified eight co-expression gene modules (Additional file 1: Fig. S7A). Importantly, we not only identified novel genes from these eight modules, but also found overlapped genes between these modules and 14 AD-related datasets (Additional file 1: Fig. S7B and Additional file 9: Table S8). These results suggest that the global gene expression alterations in human AD identified by Visium are consistent with previous findings using the bulk RNA-Seq, snRNA-Seq, and large-scale proteomic techniques, which strengthens the feasibility and reliability of implementing SRT in studying the molecular mechanisms underlying the pathogenesis of AD.

### The co-expression patterns between module-specific hub genes are altered in the presence of AD pathology

We found four modules (Yellow, Turquoise, Pink, and Brown) change along with the AD pathology. Interestingly, most of the hub genes in the yellow module are mainly expressed in oligodendrocytes based on http://www.brainrnaseq.org. Neurons and glia (microglia, astrocytes, and oligodendrocytes) interactions have been found to play important roles in the development and progression of AD pathology, especially Aβ plaques and NFTs [2, 13, 16]. The loss of co-expression of oligodendrocytes-associated hub genes in the yellow module and the lack of co-expression with hub genes in other modules support the recent findings that oligodendrocyte dysfunction may be involved in the early pathogenesis of AD [5, 16, 24]. The changes of co-expression patterns may be sufficient to induce differential alterations in biological processes critical for the prevention of neurodegeneration and neuroprotection.

To reduce technical variability and genetic background between AD and CT cases, we compared the co-expression patterns of these four gene modules in spots with AD pathology *vs* adjacent spots without AD pathology only in AD cases. We detected that the co-expression of turquoise and brown module hub genes was gradually reduced, whereas the co-expression of yellow and pink module hub genes was enhanced as the distance from Visium spots with AD pathology increases (45–245 µm) (Additional file 1: Fig. S8). The co-expression patterns, however, did not change significantly as the distance from Visium spots with both Aβ plaques and tau pathology increases (Additional file 1: Fig. S8), which might be due to the effects of both AD pathologies on hub gene co-expression being too strong to be reversed. These results demonstrate that the co-expression patterns of different gene modules are altered by AD pathology, supporting the hypothesis that interactions within and between gene modules are involved in the development and progress of AD pathology. It should also be noted that not only Aβ^+^ plaques, as previously suggested [2, 16], but also AT8^+^-NFTs/neuropil threads induce changes in the co-expression of hub genes, suggesting that altered co-expression patterns of hub genes are implicated in the pathogenesis of AD.

Differential gene expression analysis between Visium spots with AD pathology (Aβ^+^, AT8^+^, or Aβ^+^/AT8^+^) and adjacent Visium spots (level 3) without AD pathology in AD cases, identified upregulated and downregulated genes and pathways associated with each AD pathology. Interestingly, Visium spots with Aβ plaques and AT8^+^-NFTs/neuropil threads showed a significant and similar upregulation of genes and pathways associated with stress response to metal ion, detoxification and neurotransmitter uptake, and a marked downregulation of genes and pathways associated with ensheathment of neurons, protein folding, glial cell differentiation and synapse organization (Fig. 5A and Additional file 9: Table S8). This finding is consistent with recent transcriptomic, epigenomic, and proteomic analyses [12, 13, 15, 16, 24, 25, 55], highlighting the important roles of gliosis, protein folding and synaptic dysfunction in AD. Surprisingly, Visium spots with AT8^+^-NFTs/neuropil threads also exhibited upregulation of genes and pathways associated with synaptic transmission, and downregulation of genes and pathways associated with negative regulation of apoptosis. Whether NFTs cause the degeneration of neurons [66] or are actually part of a protective response that prevents neuronal death is still unclear [67]. The upregulation of synaptic transmission and the lack of enrichment of neuronal cell death and apoptosis pathways in Visium spots with AT8^+^-NFTs/neuropil threads suggest that neurons with NFTs or neighboring neurons without NFTs may exhibit a compensatory upregulation of protective mechanisms against neuronal dysfunction or death in the early stages of AD.

Our results not only validated some of the previously identified Aβ plaques [16, 17] and tangle-associated gene signatures and pathways [55], but also identified new gene signatures and pathways specific for each AD pathology (Fig. 6A). The co-expression patterns of hub genes also provided new insights for understanding the interactions between neurons and glia and how they contribute to the cellular and regional vulnerability in early AD (Fig. 5B).

### Layer- and cell type-specific DEGs associated with AD pathology

Since the diameter of a Visium spot is 55 µm, which generally includes more than one cell, we further validated DEGs associated with AD pathology at the single-cell level. Using RNAscope HiPlex smFISH assay, we first validated the DEGs that have been identified in human AD by other groups using snRNA-Seq or scRNA-Seq [13, 16, 24, 60, 61] [62–64], including *SNCG*, *STMN2*, *CSRP1, PLP1*, *GLUL*, *CD9*, *C1QB*, *SPP1*, *CD63*, *CRYAB*, and *YWHAH*. Those genes have been found to play important roles in the pathogenesis of AD [55, 63, 68–73]. Furthermore, we validated a few novel DEGs identified in this study, including *KIF5A*, *PAQR6,* and *SLC1A3*. We found that the RNA level of KIF5A was significantly increased in microglia, astrocytes, and neurons in layers II/III and V and layer V oligodendrocytes of the MTG in human AD compared to CT cases. Increased SLC1A3 in astrocytes and microglia and decreased PAQR6 in oligodendrocytes in layers II/III and V and in layer II/III astrocytes of the MTG were found in human AD compared to CT cases. The layer- and cell-type-specific role of those genes are unknown, which are warranted for future studies.

### Limitation of this study

Although the 10 × Genomics Visium platform is a powerful technique of characterizing the spatial transcriptomic profiles of rodent and human brain samples, there are a few limitations of this study. First, the Visium platform is not at single-cell resolution [16, 17], although we validated key representative genes at the single-cell level in this study. Thus, we cannot exactly provide the cell-type information for most DEGs identified in each Visium spot. Although each Visium spot is only 55 µm in diameter, the highly variable shape of cells and processes, as well as RNA diffusion from neighboring regions, suggests that information from several cells is included in each spot. Second, we performed the immunostaining of cell type-specific markers and AD pathological markers on adjacent brain sections, but not on the same section used for the H&E staining and the Visium SRT assay. Although the distance between the Visium section and the immunostaining sections is 10 or 20 µm, alignment of those sections may produce minor errors in terms of the exact location of each Visium spot in adjacent brain sections. Third, although our results were generated from similar sample size to previous publications using Visium platform [16, 74, 75], further SRT analyses of larger sample size without biological covariates (e.g. age, post-mortem interval, RNA integrity, tissue intactness and ApoE genotype), sex differences and additional functional studies are warranted for fully understanding the relationship between transcriptional changes and various AD pathology. Moreover, due to the limitation of the size of capture area of Visium GE slide, we choose to focus on one vulnerable region, the MTG, in this experiment. Future studies will include and compare specific gene expression patterns from other vulnerable or resilient brain regions. Fourth, we did not focus on particular forms of Aβ and tau aggregates in this study. Further Visium studies in combination with immunofluorescence staining of soluble *vs* insoluble forms of Aβ and tau aggregates will be needed to distinguish the transcriptional changes in the presence of various forms of AD pathology.

Overall, our Visium analysis highlights the molecular composition of the anatomical architecture of the human MTG and the complexity of glial-neuronal hub gene interactions in response to a range of AD-associated neuropathology, which may contribute to the cellular and regional vulnerability in early AD. Although we cannot distinguish drivers of degeneration from compensatory responses using post-mortem brain tissue represents end-stage disease, our spatial transcriptomic data provide insights for exploring the molecular mechanisms underlying the pathogenesis of AD and a platform for discovering potential biomarkers and disease-modifying therapeutics.

## Supplementary Information

**Additional file 1: Fig. S1 – S11**. **Fig. S1**. The distribution of AT8-positive tau pathology is similar in two adjacent serial sections from human AD MTG. **Fig. S2**. Illustration of manual layer annotation. **Fig. S3**. Illustration of generating masks for RNAscope quantification. **Fig. S4**. Validation of the sample size is sufficient for identifying conserved layer markers. **Fig. S5**. Validation of layer-specific genes on publicly available Visium SRT datasets. **Fig. S6**. Cell type deconvolution analysis of snRNA-seq data and Visium SRT data from human MTG. **Fig. S7**. Gene set enrichment analysis of gene modules identified by WGCNA in this study and 14 transcriptomics and proteomics datasets in the public domains. **Fig. S8**. Gene modules associated with AD pathology at varying spatial distances. **Fig. S9**. Representative upregulated and downregulated genes associated with AD pathology. **Fig. S10**. Validation of DEGs associated with AD pathology in layers II/III at the single-cell level using RNAscope smFISH. **Fig. S11**. Correlation between SRT gene expression and RNA counts by RNAscope.

**Additional file 2: Table S1**. Description of human post-mortem cases.

**Additional file 3: Table S2**. Overview of Visium SRT sample sequencing information and 3,000 gene features used fro SCTransform.

**Additional file 4: Table S3**. The layer-specific markers found from our human postmortem Visium SRT dataset and the previously known layer markers.

**Additional file 5: Table S4**. The proportion of spots with Aβ+, AT8+, Aβ+/AT8+ pathology which is normalized to the total number of spots in each layer from three AD cases.

**Additional file 6: Table S5**. The deconvolution analyses result using the snRNA-Seq dataset from the human MTG (Hodge et al., 2019) and the cell type proportion correlation result by comparing cell type proportion from our Visium SRT dataset with pciSeq dataset (Langseth et al., 2021).

**Additional file 7: Table S6**. The AD upregulated DEGs, downregulated DEGs, and the GO pathway analyses of those DEGs in our Visium SRT dataset. We also include the 14 published AD-related gene lists, the overlapped genes between 14 previously published AD-related gene lists and AD upregulated/downregulated DEGs identified in our Visium SRT datasets, and the Fisher exact test results in this table.

**Additional file 8: Table S7**: The AD upregulated DEGs found in our Visium SRT dataset by comparing gene expression in each layer between AD and CT, and the layer-specific AD upregulated DEGs by removing the duplicate genes among different cortical layers.

**Additional file 9: Table S8**. The gene list of eight gene modules and their GO pathway analyses in our Visium SRT dataset. We also include the comparison and Fisher Exact test results by comparing 8 gene modules with 14 published AD-related gene lists in this table.

**Additional file 10: Table S9**. The number of spots with Aβ, AT8, and Aβ/AT8 pathology, the number of their adjacent 3-level spots, the AD pathology-associated gene signatures, and GO pathway analyses corresponding to each pathology are shown in this table.

## Data Availability

The raw sequencing data and unfiltered UMI count matrices are available on the Gene Expression Omnibus (GEO) under accession code GSE220442. All cloupe files, data and analysis are also available at our in-house scREAD database at https://bmbls.bmi.osumc.edu/scread/stofad-2.
