# Supplementary material for: Spatially resolved transcriptomics reveals genes associated with the vulnerability of middle temporal gyrus in Alzheimer’s disease
Source: Acta Neuropathol Commun. 2022 Dec 21;10:188. doi: 10.1186/s40478-022-01494-6 (PMC9773466; doi:10.1186/s40478-022-01494-6)
Supplement: Supplementary file 1 — Additional file 1: Fig. S1 – S11. Fig. S1. The distribution of AT8-positive tau pathology is similar in two adjacent serial sections from human AD MTG. Fig. S2. Illustration of manual layer annotation. Fig. S3. Illustration of generating masks for RNAscope quantification. Fig. S4. Validation of the sample size is sufficient for identifying conserved layer markers. Fig. S5. Validation of layer-specific genes on publicly available Visium SRT datasets. Fig. S6. Cell type deconvolution analysis of snRNA-seq data and Visium SRT data from human MTG. Fig. S7. Gene set enrichment analysis of gene modules identified by WGCNA in this study and 14 transcriptomics and proteomics datasets in the public domains. Fig. S8. Gene modules associated with AD pathology at varying spatial distances. Fig. S9. Representative upregulated and downregulated genes associated with AD pathology. Fig. S10. Validation of DEGs associated with AD pathology in layers II/III at the single-cell level using RNAscope smFISH. Fig. S11. Correlation between SRT gene expression and RNA counts by RNAscope. [file 40478_2022_1494_MOESM1_ESM.docx]

**Supplementary figures and legends**


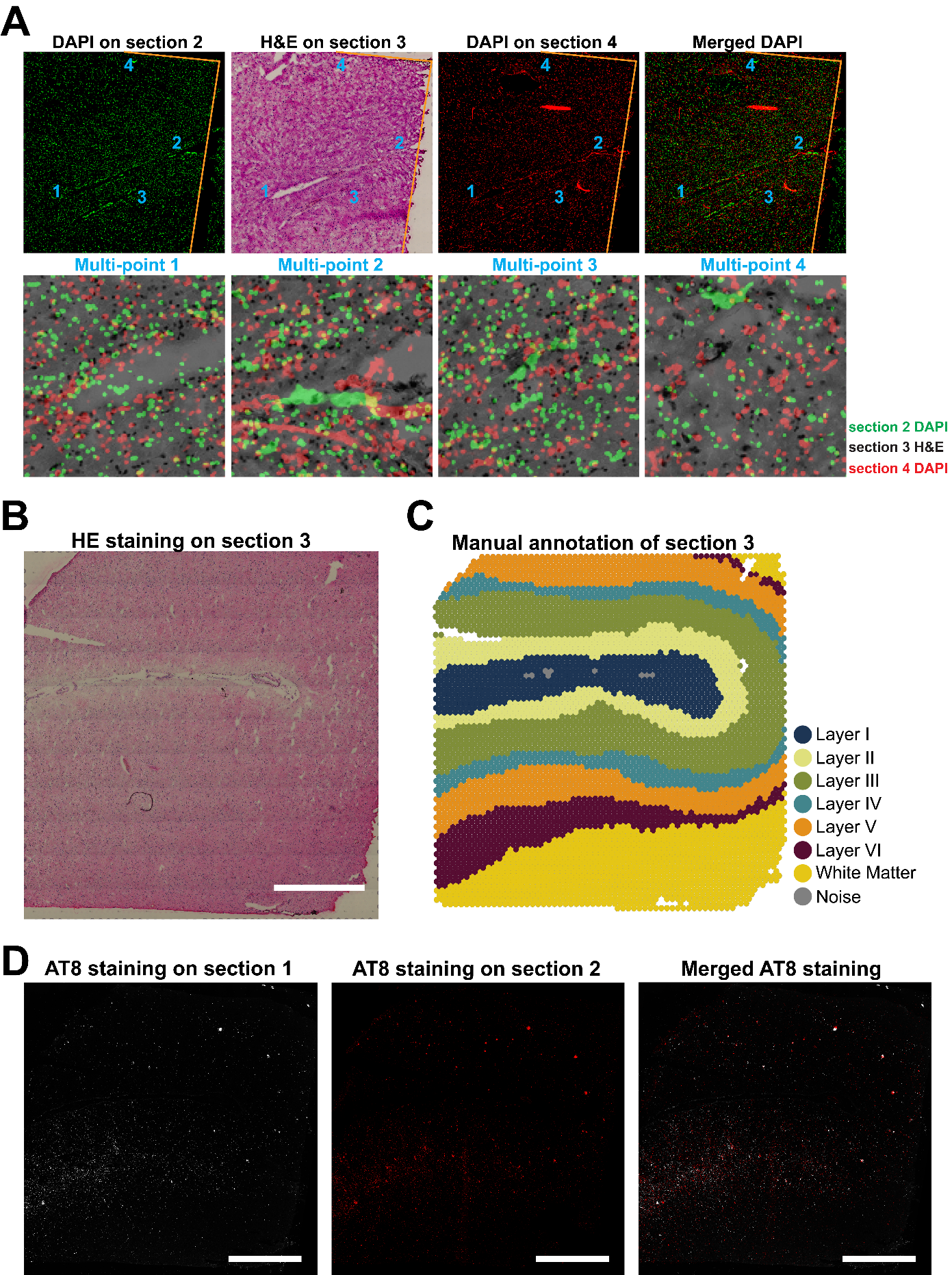


**Fig. S1. The distribution of AT8-positive tau pathology is similar in two adjacent serial sections from human AD MTG.**

(**A**) Illustration of adjacent section alignment based on the outline of sections, DAPI staining, and brain architecture. (**B**) The H&E staining and (**C**) the manual annotation of Visium spots on the section No.3 of AD-3 sample. (**D**) Two adjacent serial sections (10 µm) from the MTG of AD-3 at Braak stages III/IV were immunostained with AT8 (pS202/T205 tau) antibody. The AT8+ ptau staining was enriched in layers II/III and V of both sections with close proximity between the staining in section 1 (white) and section 2 (red), which is shown by the merged image. Scale bar = 2 mm.


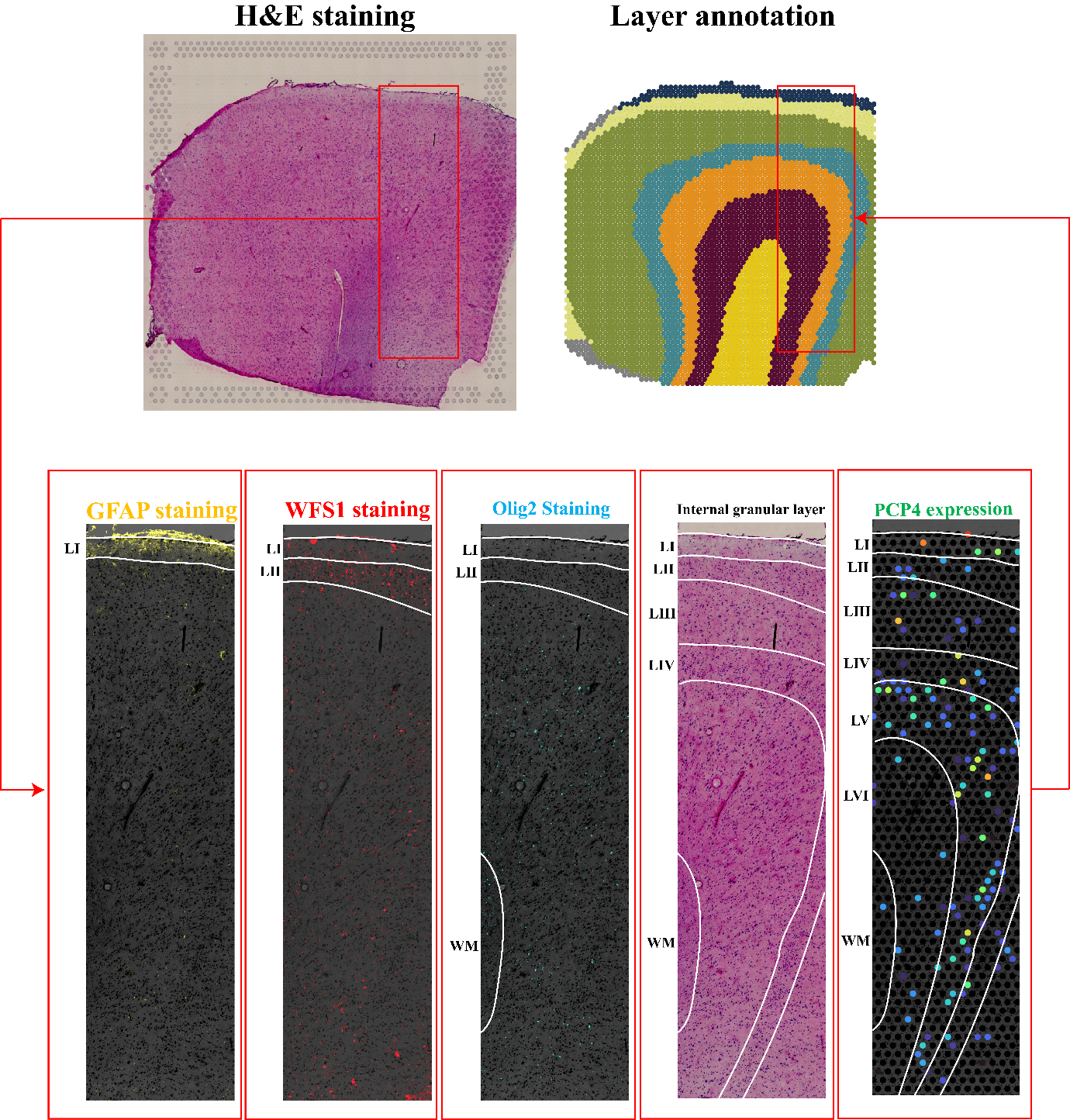


**Fig. S2. Illustration of manual layer annotation.**

Cortical layers and the WM of human MTG were manually annotated by GFAP staining (yellow), WFS1 staining (red), Olig2 staining (light blue), H&E staining, and PCP4 gene expression level (see details in Methods).


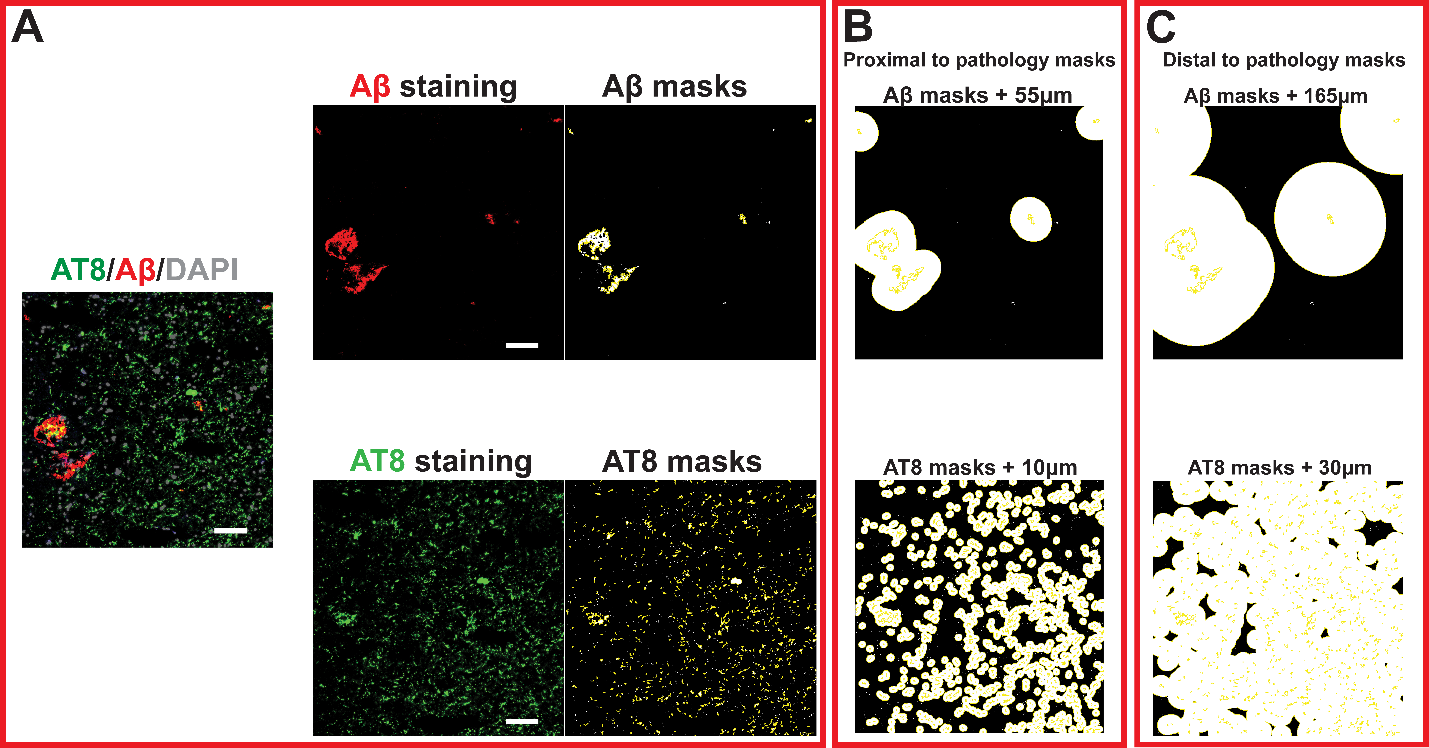


**Fig. S3. Illustration of generating masks for RNAscope quantification**

(**A**) Aβ and AT8 staining were imported into ImageJ separately and converted into “pathology-masks” (see detail in Methods). (**B**) Pathology-masks were extended 55µm from the edge to generate “Proximal to pathology” masks. All the circled cells within the masks (white parts) will be assigned as “proximal to pathology”. (**C**) Pathology-masks were further extended 165µm from the edge to generate “distal to pathology” masks. All the circled cells outside of the masks (black parts) will be assigned as “distal to pathology”. Scale bar, 100µm.


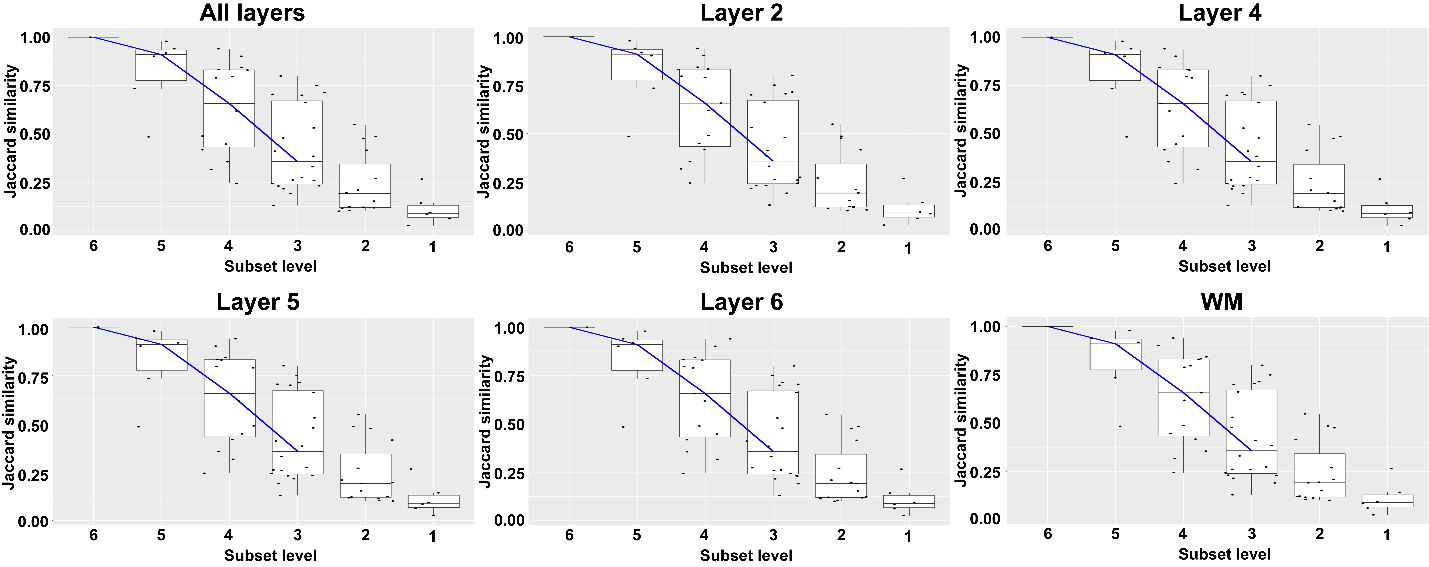


**Fig. S4. Validation of the sample size is sufficient for identifying conserved layer markers.**

The Jaccard/Tanimoto similarity test was performed to explore the power of six samples for identifying conserved layer markers for all layers, layer II, layer IV, layer V, layer VI, and the white matter (WM). The x-axis is the number of subset level. For example, “5” means subsetting five samples from six samples. Therefore, there are six subset levels (corresponding to six points in the boxplot). The y-axis is the Jaccard similarity score to indicate the similarity between identified markers from the subsetting samples and all samples.


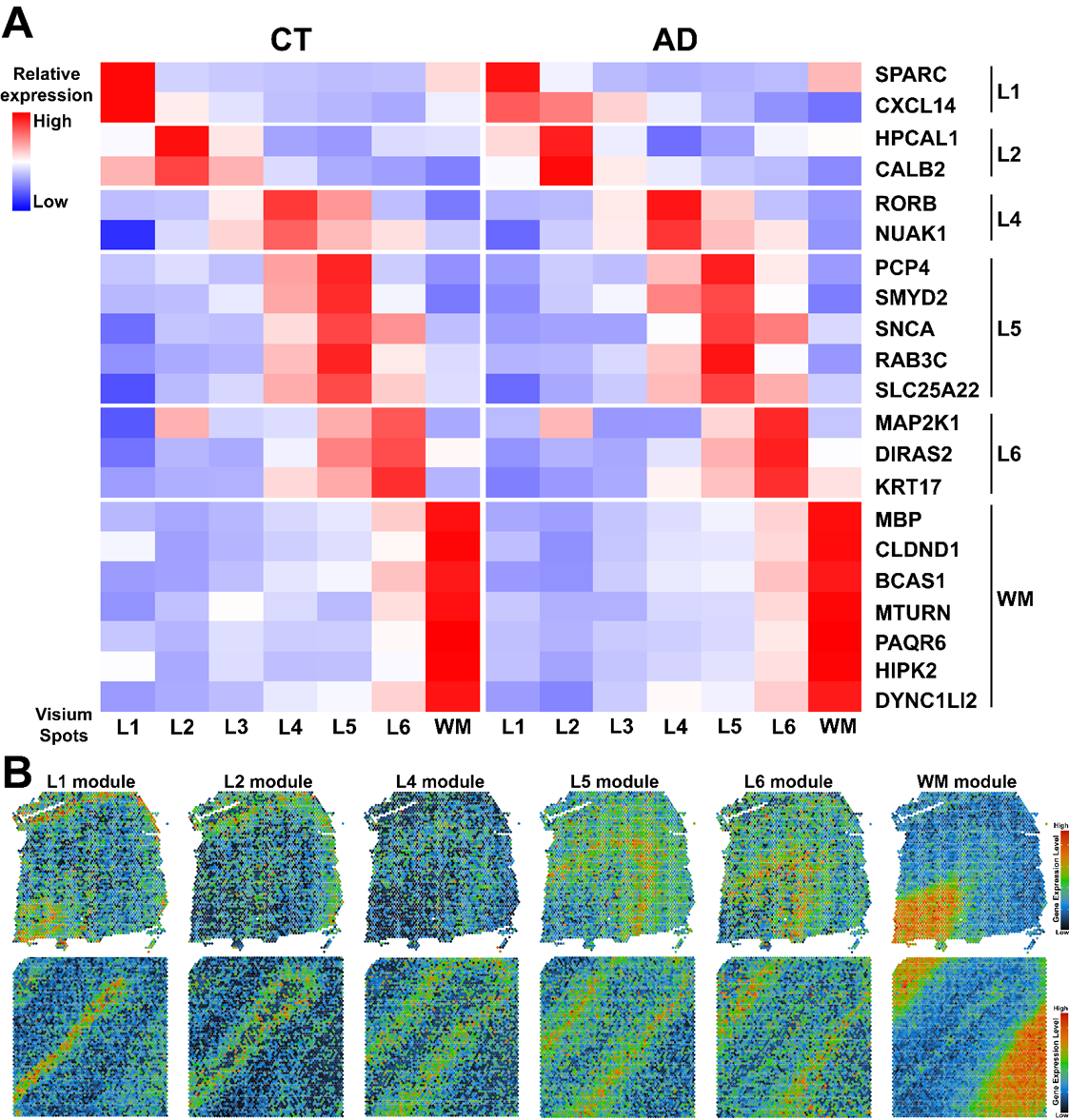


**Fig. S5. Validation of layer-specific genes on publicly available Visium SRT datasets.**

(**A**) Heatmap validation of the layer-specific gene modules in CT and AD samples separately. The red color indicates a relevantly higher expression of the gene than other layers, while the blue color indicates a lower expression. (**B**) Spatial maps show the gene module score for each layer-specific marker on the human brain sample (Sample ID: 151673) from another group (top panel), and 10x Genomics’ public dataset (Sample ID: Human brain 2) (bottom panel). The red color represents a higher score, indicating the average gene expression value of the gene module higher than random control genes. Conversely, the black color represents a lower score, indicating the average gene expression value of the gene module lower than random control genes. Letter “L” represents “layer”.


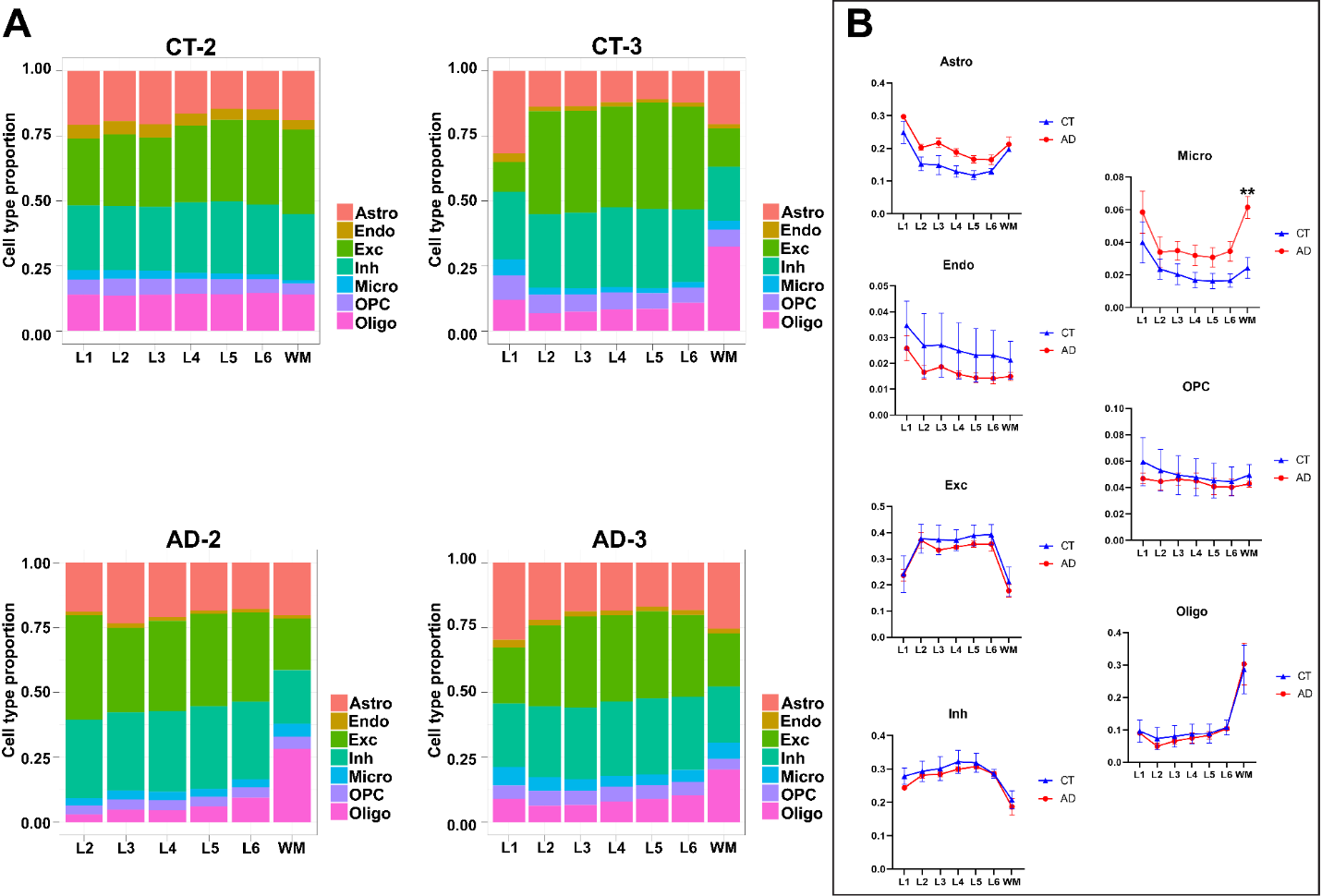


**Fig. S6. Cell type deconvolution analysis of snRNA-seq data and Visium SRT data from human MTG.**

(**A**) The cell-type-specific distributions of seven main cell types (see details in Methods) among six cortical layers and the adjacent WM of three CT (CT-1, -2, -3) and three AD (AD-1, -2, -3) human MTG were deconvoluted using Cell2location (see details in Methods). Stack bar plots show the average proportion of 7 cell subpopulations in layers I-VI (L1-L6) and the adjacent WM. Astro, astrocytes; Endo, endothelial cells; Exc, excitatory neurons; Inh, inhibitory neurons; Micro, microglia; Oligo, oligodendrocytes; and OPC, oligodendrocyte precursor cells. (**B**) The cell type proportions in each layer of CT and AD samples were compared using two-way ANOVA in Prism 9.2.0. The cell type proportions did not significantly change in each cortical layer and the WM of AD, except microglia which significantly increased (log2 fold change = 1.336, p-value = 0.007) in the WM of AD compared to CT.


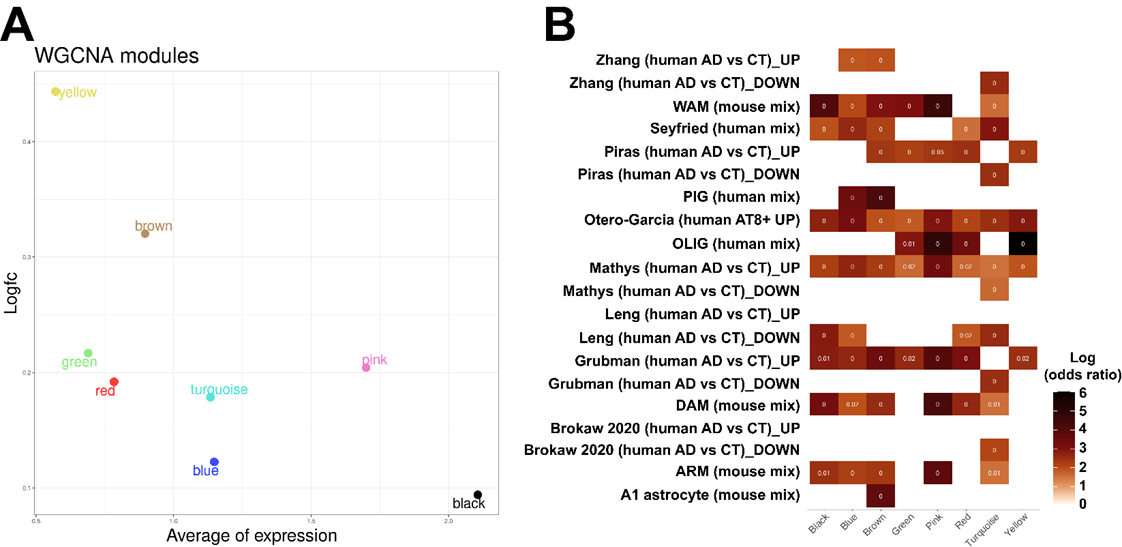


**Fig. S7. Gene set enrichment analysis of gene modules identified by WGCNA in this study and 14 transcriptomics and proteomics datasets in the public domains.**

(**A**) The weighted gene co-expression network analysis (WGCNA) of 10,000 highly variable genes from six samples identified eight gene modules. The MA plot shows the mean expression of eight gene modules against log fold change (module mean expression from AD spots versus that from CT spots). (**B**) The Fisher’s exact test between 8 gene modules and 14 previously published transcriptomic and proteomics datasets (**Table S8**). The x-axis and y-axis indicate objects of Fisher’s exact test. Heatmap block color indicates the log-transformed odds ratio. The digit number in the middle of the heatmap block indicates the adjusted p-value. If the heatmap block has the non-significant adjusted p-value, the block color will be assigned as white.


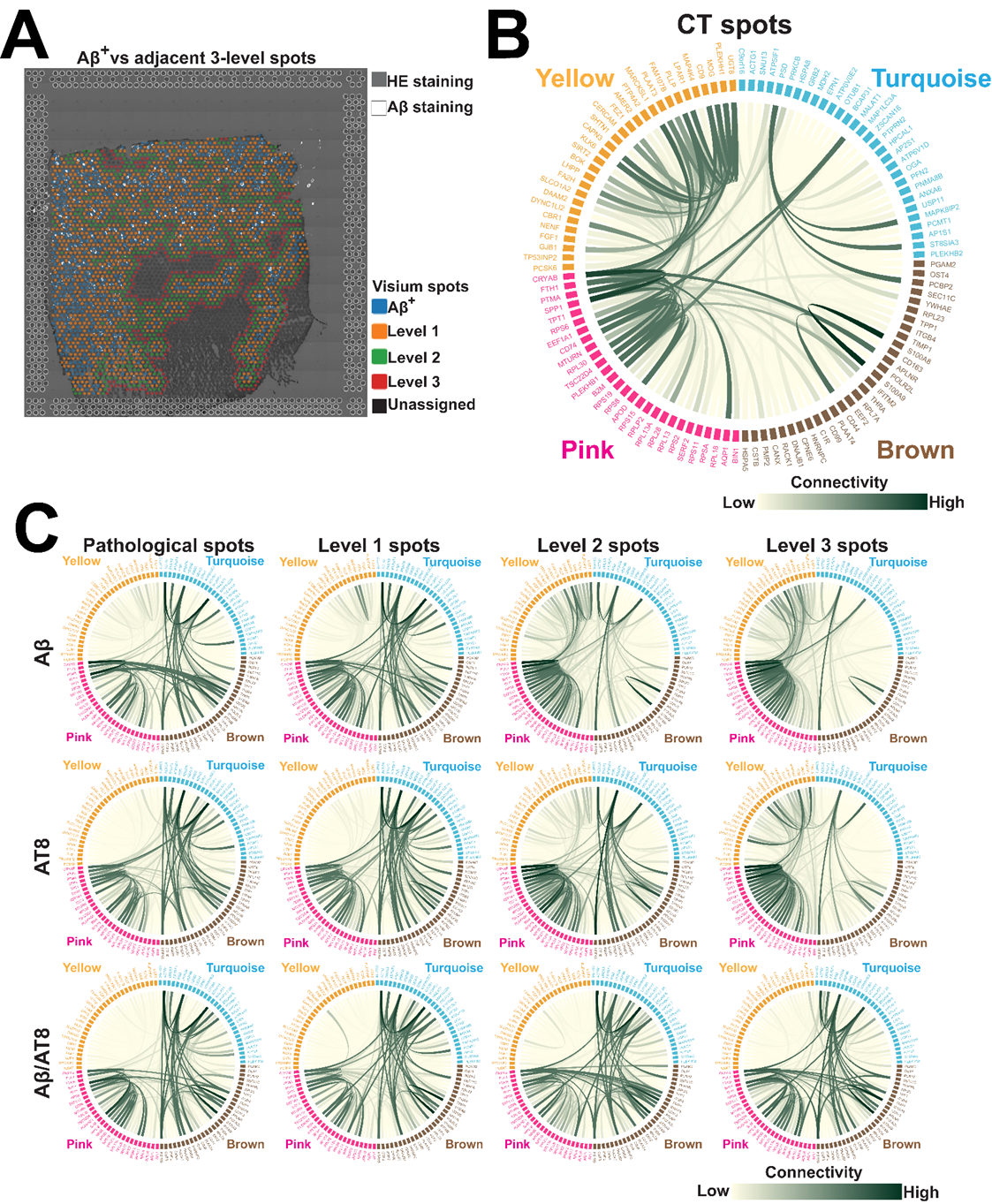


**Fig. S8. Gene modules associated with AD pathology at varying spatial distances.**

(**A**) Aβ plaques were stained with human specific Aβ antibody using IF and aligned to Visium slide. Spots with Aβ+ staining were labeled as Aβ+ Visium spots using Loupe Browser. Spot levels (1-3) without AD pathology were assigned based on their distance (level 1: 45 µm, level 2: 145 µm, and level 3: 245 µm) from the site of pathology. (**B**) Co-expression of four gene modules (Yellow, Turquoise, Pink and Brown modules) in CT spots based on the WGCNA analysis. (**C**) Co-expression of these four modules in AD human MTG at sites of AD pathology (Aβ+, AT8+, or Aβ+/AT8+) vs. surrounding Visium spots (levels 1-3) without AT8+ or Aβ+ staining.


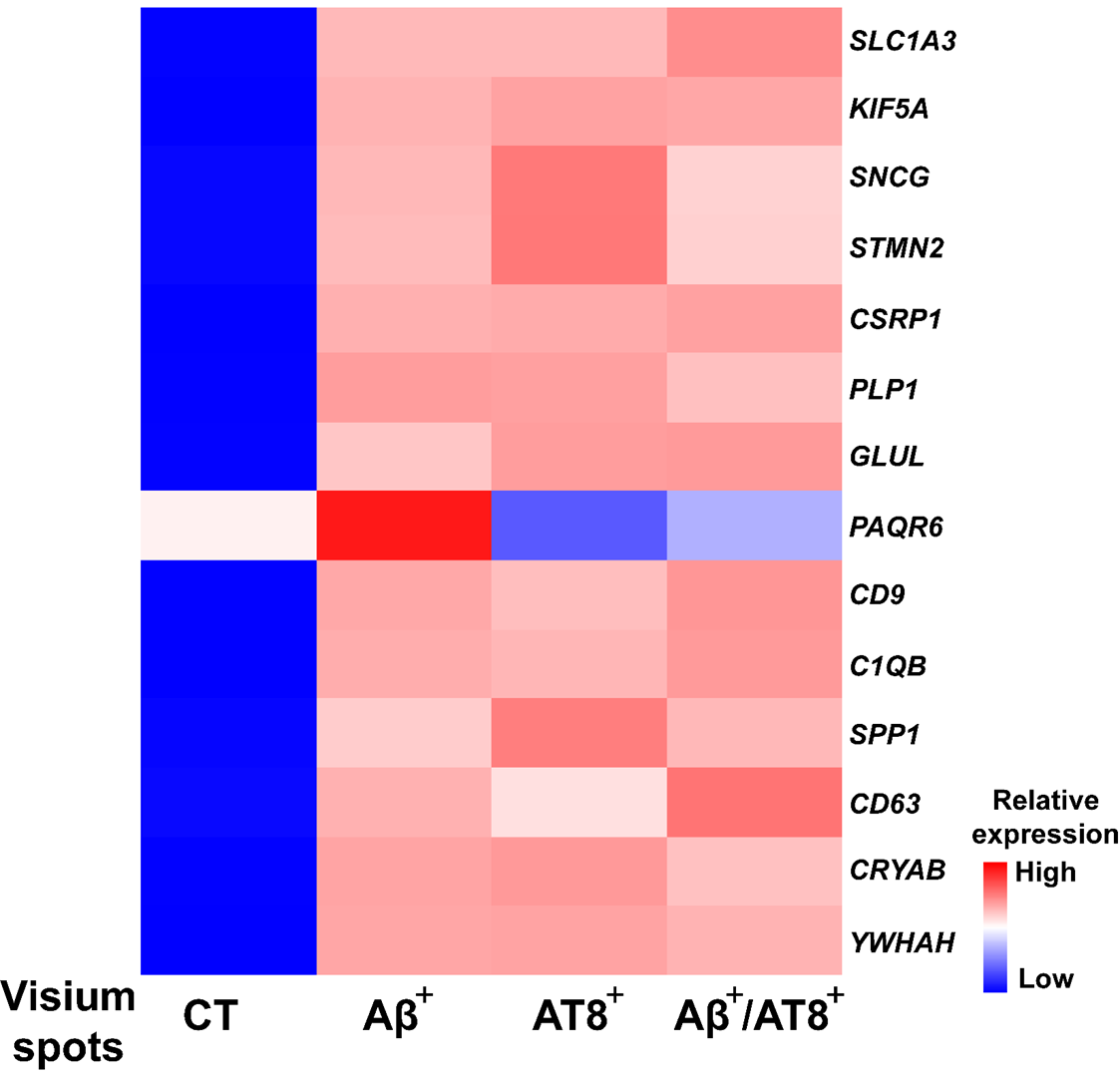


**Fig. S9. Representative upregulated and downregulated genes associated with AD pathology.**

Heatmap of Z-scores for 14 representative DEGs associated with different AD pathology (Aβ+, AT8+, and Aβ+/AT8+) and the control (CT) in three AD and three CT Visium samples. These 14 DEGs were selected as probes for cell-type-specific validation in AD (Braak stages III/IV) and CT samples using RNAscope single-molecule fluorescent in situ hybridization (smFISH). The red color indicates a relevantly higher expression of that gene in particular spots than other spots, while the blue color indicates a lower expression.


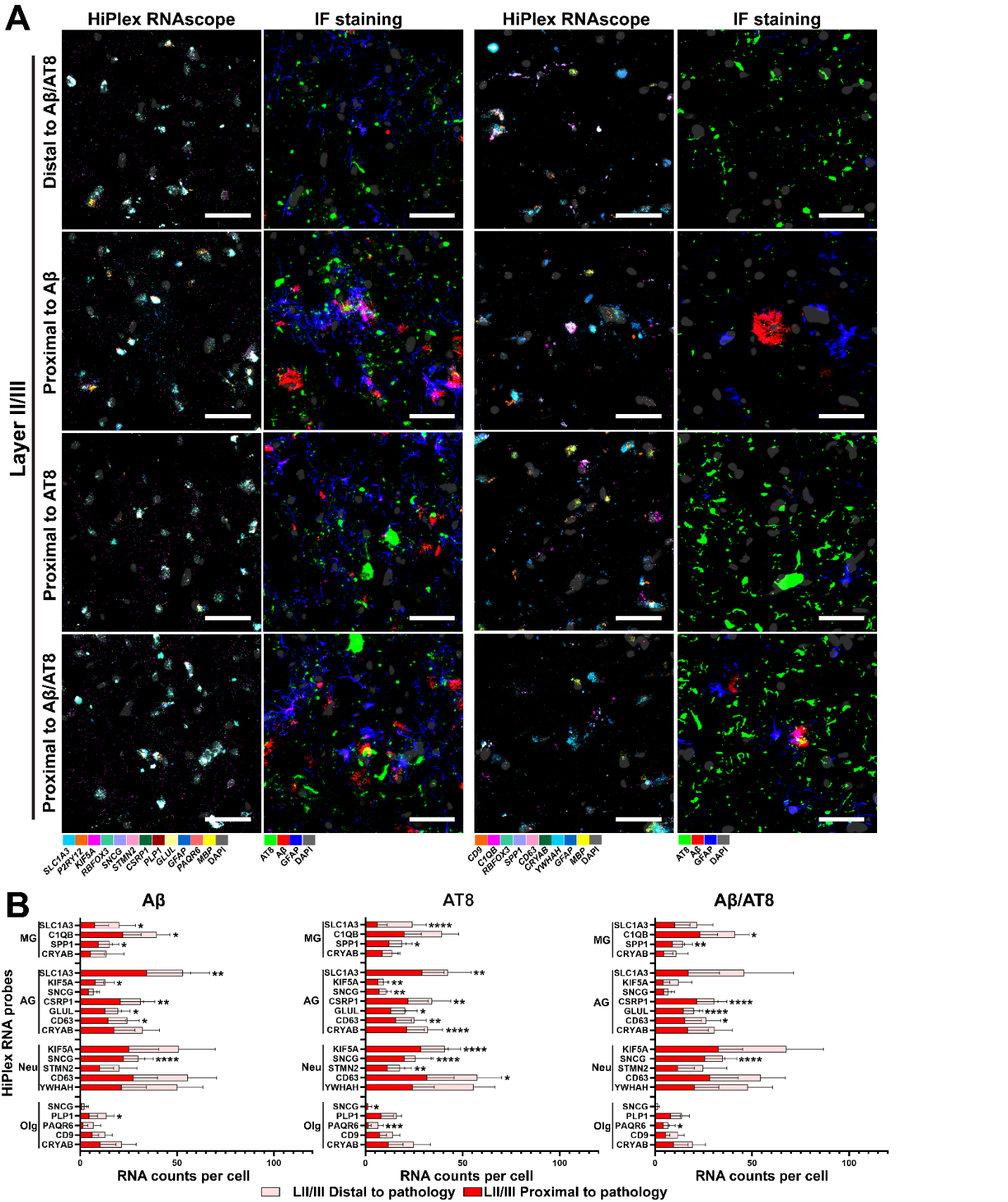


**Fig. S10. Validation of DEGs associated with AD pathology in layers II/III at the single-cell level using RNAscope smFISH.**

(**A**) Representative RNAscope images (left panel) and their corresponding post-IF staining of Aβ, AT8, and GFAP (right panels) in layer II/III distal to and proximal to pathological spots. All images from the same cells were aligned and registered by imageJ (see details in Methods). (**B**) RNAscope probes against human SLC1A3, KIF5A, SNCG, STMN2, CSRP1, PLP1, GLUL, PAQR6, CD9, C1QB, SPP1, CD63, CRYAB, and YWHAH in microglia (P2RY12+/C1QB+), astrocytes (GFAP+), neurons (RBFOX3+), and oligodendrocytes (MBP+) were quantified and compared within three AD cases. For quantification, we identified 20~50 microglia, astrocytes, and oligodendrocytes, and 60~90 neurons from each case (see details in Methods). * P< 0.05, ** P<0.01, *** P<0.001, **** P<0.0001 (Mann-Whitney test, proximal to pathology vs distal to pathology). Scale bar, 50 µm. See high-resolution RNAscope images at: https://bmbls.bmi.osumc.edu/scread/stofad-2.


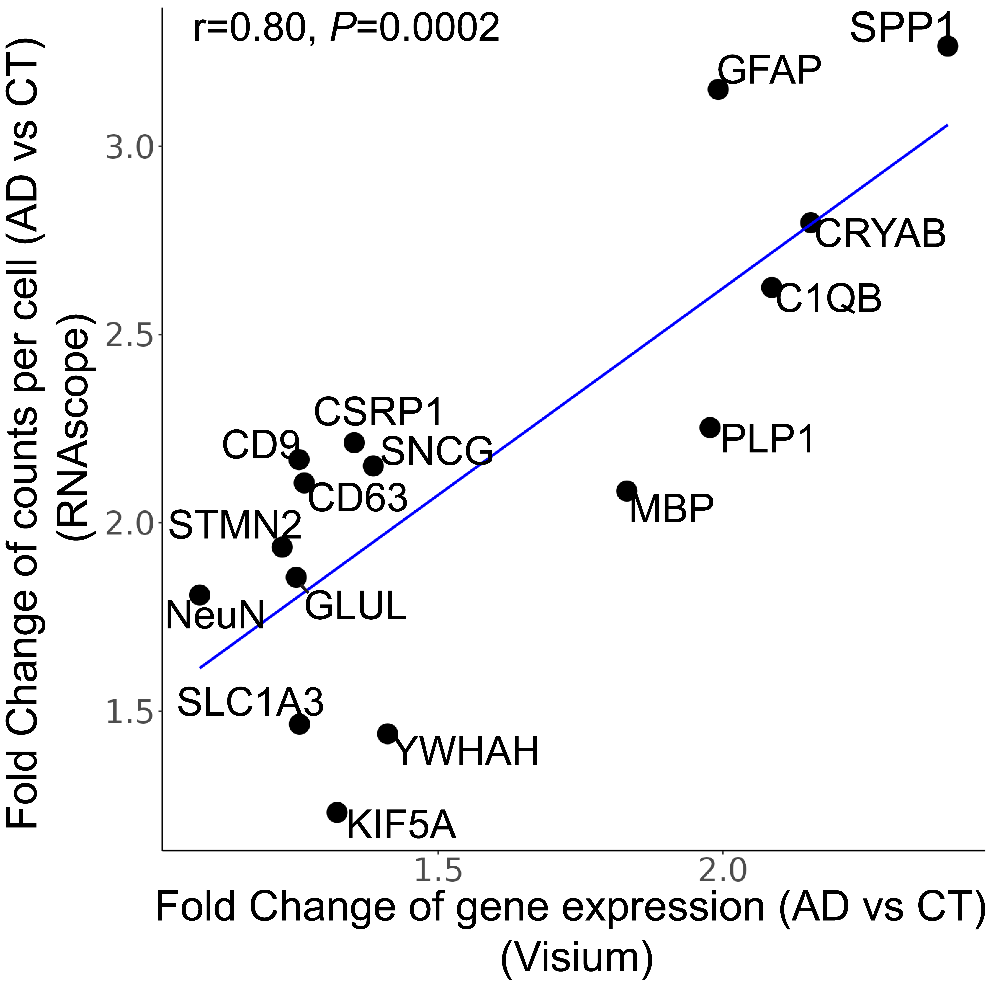


**Fig. S11. Correlation between SRT gene expression and RNA counts by RNAscope.**

The plot reflects correlations of gene expression calculated by RNAscope and Visium SRT. Each spot represents a selected gene. The x-axis represents the fold change of gene expression between AD and control samples measured by Visium. The y-axis represents the fold change of probes between AD and control spots detected by RNAscope.
