# Supplementary material for: Spatially resolved transcriptomics reveals genes associated with the vulnerability of middle temporal gyrus in Alzheimer’s disease
Source: Acta Neuropathol Commun. 2022 Dec 21;10:188. doi: 10.1186/s40478-022-01494-6 (PMC9773466; doi:10.1186/s40478-022-01494-6)
Supplement: Supplementary file 2 — Additional file 2: Table S1. Description of human post-mortem cases. [file 40478_2022_1494_MOESM2_ESM.docx]

**Table S1. Description of human post-mortem cases.**

| **Case ID** | **Sex** | **Age (yr)** | **AD** | **Other NDs** | **MMSE before death** | **Amyloid Thal phase (A)** | **Braak NFT stage (B)** | **CERAD neuritic plaque score (C))** | **Section type** | **Use** |
| --- | --- | --- | --- | --- | --- | --- | --- | --- | --- | --- |
| CT-1 | M | 58 | No | No | n/a | 0 | 1 | 0 | FR | Visium, RNAscope |
| CT-2 | M | 72 | No | No | 29 | 0 | 1 | 0 | FR | Visium |
| CT-3 | M | 82 | No | No | 30 | 0 | 1 | 0 | FR | Visium |
| AD-1 | M | 86 | Yes | No | 20 | 3 | 2 | 3 | FR | Visium |
| AD-2 | M | 86 | Yes | No | 19 | 3 | 2 | 3 | FR | Visium |
| AD-3 | M | 89 | Yes | No | 25 | 3 | 2 | 2 | FR | Visium, RNAscope |
| CT-4 | M | 82 | No | No | 29 | 0 | 1 | 0 | FR | RNAscope |
| CT-5 | F | 53 | No | No | n/a | 0 | 1 | 0 | FR | RNAscope |
| CT-6 | F | 75 | No | No | 28 | 0 | 1 | 0 | FR | RNAscope |
| AD-4 | M | 90 | Yes | No | n/a | 2 | 2 | 2 | FR | RNAscope |
| AD-5 | F | 98 | Yes | No | 22 | 3 | 2 | 2 | FR | RNAscope |
| AD-6 | F | 90 | Yes | No | 24 | 3 | 2 | 3 | FR | RNAscope |

CT, Control; AD, Alzheimer’s disease; M, male; F, female; NDs: neurodegenerative diseases including Parkinson’s disease, Dementia with Lewy bodies, Vascular dementia, Progressive supranuclear palsy, Hippocampal sclerosis, Dementia lacking distinctive histology, Motor neuron disease, Corticobasal degeneration, Pick’s disease, Huntington’s disease, Multiple system atrophy, Argyrophilic grain disease, Cerebral white matter rarefaction, Frontotemporal lobar degeneration with TDP-43, and Multiple sclerosis; MMSE, Mini-Mental State Exam; All samples are classified according to the ABC scoring method described in the National Institute of Aging-Alzheimer’s Association guidelines for the neuropathologic assessment of AD (NIA-AA AD) (Montine *et al*). FR, frozen section.

**Reference**
Montine, T. J. et al. National Institute on Aging-Alzheimer's Association guidelines for the neuropathologic assessment of Alzheimer's disease: a practical approach. Acta Neuropathol 123, 1-11, doi:10.1007/s00401-011-0910-3 (2012).
